# Resilience of health systems in Africa to infectious disease shocks: A qualitative evidence synthesis

**DOI:** 10.1371/journal.pgph.0006938

**Published:** 2026-08-07

**Authors:** Denis Okethwangu, Sherry Ahirirwe, Mahima Venkateswaran, Roy Mayega, Elizeus Rutebemberwa, Felix Ocom, Victoria Nankabirwa, Charles L. Okot, Evelyne B. Nyachwo, Sandra Nabatanzi, Evelyn Asio, Umaer N. Mohammed, Alex R. Ario, Frode Forland, Suzanne N. Kiwanuka

**Affiliations:** 1 Department of Health Policy, Planning and Management, Makerere University School of Public Health, Kampala, Uganda; 2 Uganda National Institute of Public Health, Ministry of Health, Kampala, Uganda; 3 Department of Global Health, Norwegian Institute of Public Health, Oslo, Norway; 4 Department of Epidemiology and Biostatistics, Makerere University School of Public Health, Kampala, Uganda; 5 Resilience Africa Network, Makerere University School of Public Health, Kampala, Uganda; 6 Epidemic Preparedness and Response Unit, World Health Organization Regional Office for Africa, Brazzaville, Congo; 7 Department of Disease Control and Environmental Health, Makerere University School of Public Health, Kampala, Uganda; 8 Seconded Expert, Africa Centers for Disease Control and Prevention, Addis Ababa, Ethiopia; Institute of Development Studies, UNITED KINGDOM OF GREAT BRITAIN AND NORTHERN IRELAND

## Abstract

Strong and resilient health systems are those equipped to withstand shocks and continue providing quality services as response measures are implemented. We conducted a qualitative evidence synthesis of stakeholders’ understanding of health system resilience in Africa, focusing on definitions and attributes. We searched for peer-reviewed articles and grey literature, filtered for Africa, from 1980 to November 2025, using the SPIDER framework. The search was conducted in four databases: PubMed, the Bielefeld Academic Search Engine, the Cumulative Index to Nursing and Allied Health Literature, and Scopus; we also reviewed the websites of the World Health Organization (WHO), Africa Centers for Disease Control and Prevention (Africa CDC), and Ministries of Health of African countries. Articles were selected based on predefined inclusion and exclusion criteria. Qualitative articles were appraised using the Critical Appraisal Skills Programme, and mixed-methods articles using the Mixed Methods Appraisal Tool. We mapped the distribution of included articles by the country studied, the infectious disease responsible for the shock, and the health system building block described. For each article, we identified the definition of health system resilience and its attributes. The search yielded 5,448 relevant articles, eighty-three of which were included in the synthesis. Articles were from 48 of the 54 African countries. Up to 74.5% of the articles focused on COVID-19; others were on Ebola Virus Disease, cholera, and meningitis. Service delivery, leadership and governance and health workforce were the most frequently cited health system building blocks. Adaptive capacity of health systems resilience was most frequently cited, followed by absorptive capacity, preparedness, and recovery. Identified attributes of a resilient health system were community engagement and involvement; leadership and governance; collaborations and partnerships; human resources for health; health education and promotion; health information systems; health service delivery; decentralization and local governance; health infrastructure and logistics; preparedness; learning and adaptation; and innovation and financing.

## Introduction

The normal functioning of health systems can be disrupted by significant shocks, primarily due to the pressures they place on various components of the system [1]. Documented shocks that may affect health systems include infectious disease outbreaks, large-scale population displacements, civil unrest, and climate-related issues [2–4]. These shocks diminish a health system’s ability to provide high-quality routine healthcare and to respond effectively to emergencies. Disruptions within health facilities frequently occur due to the reallocation of resources, whether human, financial, or material, to crisis response efforts, thereby diverting resources from service delivery, logistics, or influencing community perceptions [3]. Subsequently, the health system is left overwhelmed and unable to function normally. On a larger scale, systemic issues, including governance deficiencies, insufficient financing, structural and resource limitations, and fragile information infrastructure, further intensify disruptions [5]. Such disruption may result in loss of life from other illnesses, economic downturns, social upheavals, and diminished access to quality services in specialized clinics (e.g., non-communicable diseases, reproductive health, and HIV), which are frequently deprioritized during outbreaks [6,7]. This inability of health systems to provide essential health services during response to shocks directly impacts universal health coverage [8]. The COVID-19 pandemic exposed the insufficiency of universal health coverage, which is defined as: 1) an adequate number of trained health professionals; 2) availability of medicines; 3) resilient health information systems, including surveillance mechanisms; 4) suitable infrastructure; 5) adequate public financing; and 6) a robust public sector capable of providing equitable and high-quality services [9,10]. The increasing frequency of outbreaks of emerging and re-emerging infectious diseases further undermines global health security and the attainment of universal health access. In low-resource settings, especially in Africa within the meningitis belt and arbovirus-endemic regions, outbreaks are frequent [11,12]. Since 2020, the African region has faced more than 100 major outbreaks annually. Uganda alone experienced over 800 disease outbreaks between 2000 and 2022 [13,14]. Notably, a 2025 review reported 85 outbreaks between 2017 and 2022, most of which were vaccine-preventable diseases (VPDs) and viral hemorrhagic fevers (VHFs) [14–16]. Health systems in Africa, however, continue to lack sufficient capacity to respond effectively to emergencies while maintaining routine operations and care [15,17]. Resilience is defined as “the capacity of health actors, institutions, and populations to prepare for and effectively respond to crises; maintain core functions when a crisis hits; and, informed by lessons learned, reorganize if conditions require it” [18]. The concept gained prominence during the Ebola Virus Disease outbreak in West Africa during 2014–2016, when health systems lost the ability to ensure health for all and achieve good outcomes [19]. Similarly, the COVID-19 pandemic revealed extensive challenges in establishing resilient systems [20–22]. Resilience has often been narrowed to emergency response rather than the broader aspects that sustain essential services, including financing, human resources, and community engagement [23].

To establish resilient health systems globally, it is essential to contextualize the concept of resilience, as the current lack of a shared understanding hampers its effective operationalization for system strengthening. For Africa, this necessitates analyzing the concept through the perspectives of various local stakeholders. We conducted a qualitative evidence synthesis of published literature indexed in prominent databases and grey literature. Our primary objective was to examine evidence regarding health system resilience in African nations, with aims of synthesizing the interpretations of resilience and identifying characteristics of resilient systems in response to infectious disease shocks.

## Materials and methods

### Search and screening

Our evidence synthesis was ted in accordance with the PRISMA Reporting Guidelines [24] (S1 Checklist).

We developed a stepwise search strategy using keywords in our review protocol, initially in PubMed, and adapted it for the other databases.

Search #1: resilience
Search #2: health system OR healthcare systems
Search #3: pandemics OR disease outbreaks
Search #4: Africa
Search #5: study type
Search #6: health systems response to disease outbreaks in Africa—#2 AND #3 AND #4
Search #7: Qualitative and mixed studies on health systems response to disease outbreaks in Africa—#6 AND #5
Complete search (#8): #7 AND #1

For search #4, we adopted the African continent search filter developed by Pienaar et al. [25]. However, because the filter was published before Swaziland changed its name to Eswatini, we manually updated it to include the new name.

The development of the research question was guided by the SPIDER framework, an alternative to the Population, Intervention, Comparison, and Outcome (PICO) framework for guiding search strategies in qualitative and mixed-methods reviews [26]. The SPIDER framework was operationalized by translating each of its components: Sample (S), Phenomenon of Interest (PI), Design (D), Evaluation (E), and Research type (R), into distinct search blocks. For each component, we identified relevant keywords, synonyms, and controlled vocabulary terms. These terms were developed iteratively through preliminary searches and refinement of key concepts. Additional terms were generated using the PubMed exploded medical subject headings (MeSH) function. The Sample block included terms related to African settings; the Phenomenon of Interest block captured concepts of health system resilience and infectious disease shocks; the Design and Research type blocks included terms reflecting qualitative and mixed methods approaches; and the Evaluation block focused on terms related to definitions, descriptions, and conceptualizations. The structure of these blocks was adapted across databases to account for differences in indexing systems and search functionalities. Consequently, our research question was: “How is health system resilience to infectious disease shocks in Africa defined and described in qualitative and mixed-methods studies?” (Table A in S1 Appendix).

We developed blocks using the Boolean operator “OR” to capture variations in terminology and “AND” to combine the resulting SPIDER blocks, ensuring that retrieved records addressed all key aspects of the research question. Truncations and phrase searching were applied, where appropriate, to increase sensitivity while maintaining specificity. We combined the different searches to derive the complete set of search terms and results.

We adopted the search terms from PubMed for the other databases, depending on the limits on number of search terms and unique search features. Other electronic databases we searched were the Bielefeld Academic Search Engine (BASE), Medline, the Cumulative Index to Nursing and Allied Health Literature (CINAHL), and Scopus.

We conducted searches between November 2023 to November 2025. PubMed, NLM (pubmed.ncbi.nlm.nih.gov/) was initially searched on November 20, 2023; CINAHL accessed through EBSCO was searched on February 28, 2024; Scopus accessed through Elsevier was searched on February 28, 2024; and BASE (www.base-search.net) was searched on March 20, 2024. Searches for all the databases were updated on November 14, 2025. We also explored the websites of organizations such as the World Health Organization (www.who.int/), the US Centers for Disease Control and Prevention (www.cdc.gov), and the Africa Centers for Disease Control and Prevention (africacdc.org) for grey literature. Additional articles were identified from the reference lists of the selected articles (Table B in S1 Appendix).

Search results were managed using a multi-platform workflow to optimize different stages of the review process. Records were first imported into Zotero, where duplicates were removed using its structured deduplication function [27]. The deduplicated dataset was then transferred to Rayyan for title and abstract screening, where its interface facilitated initial screening and tagging of records [28]. The first author (DO) and second author (SA) conducted blinded title and abstract screening. Where there was a disagreement about whether to include or exclude a record, a decision was made by consensus. Following this stage, studies meeting inclusion criteria were exported and imported into Covidence software for full-text screening. Covidence was selected for this stage due to its more advanced functionality for managing full-text review and study selection [29]. Articles where full texts could not be retrieved were excluded from the full-text screening. For such articles, we sent emails to the contact address of the first or corresponding author requesting the full texts. We excluded them only after we had not received a response for 4 weeks. Authors DO and SA independently reviewed the full texts, including or dropping articles based on the inclusion and exclusion criteria.

### Inclusion and exclusion criteria

Peer-reviewed journal articles reporting primary data on health system resilience to infectious diseases, published from 1980 onward, were included. The cut-off year was selected to include any earlier studies on resilience in the health system, even though the concept took root in the health sector in the early 2000s. Our inclusion criteria were: 1) Articles on health system resilience to infectious disease shocks; 2) Studies conducted in an African country; and 3) Articles that are qualitative or mixed methods studies. Articles were included if they met these criteria and were published from 1980 onward. We excluded articles that examined the resilience of health systems to shocks other than infectious diseases (e.g., conflict or climate change) and reviews. We also excluded quantitative articles, opinions, commentaries, and editorials because of lacking primary qualitative data to support interpretive synthesis (S1 Table). We also included full-text articles written in other languages [30,31]. Non-English articles were translated using the Translate function in Microsoft Edge [32].

### Data extraction

Our data extraction was conducted in Covidence [29]. The data extraction tool was developed in MS Excel by DO, with technical reviews from MV, AAR, and SNK. In addition to the bibliometric data (title of the article, author, and year of publication), we also extracted data on the country or countries of study, the definition or description of health system resilience, the characteristics and attributes of a resilient health system, and the shocks studied. Data extraction was also conducted independently DO and SA. A consensus meeting was used to resolve disagreements during data extraction.

### Quality assessment of reports

Articles selected from full-text screening were assessed for quality using the Critical Appraisal Skills Programme (CASP) checklist for qualitative studies, while for mixed-methods studies, we used the Mixed Methods Appraisal Tool (MMAT) checklist [33,34]. These two checklists were uploaded into the Covidence software to facilitate the appraisal process. The CASP checklist systematically evaluates three broad areas in a 10-question format. These issues include: 1) Are the results of the study valid (questions 1–6)? 2) What are the results? 3) Will the results be applicable locally? Each question offers three response options: “yes”; “can’t tell”; and “no”. Prompts, also known as hints, help guide responses to each question. To quantitatively assess the quality of the articles, we assigned a score of 1 to every “yes” score; 0 to “can’t tell”; and -1 to “no”. This was a modification to a study that assigned 2 to every “yes” response; 1 to “can’t tell”; and 0 to “no” [35]. Based on total scores, studies were stratified into three quality tiers: high (7–10), moderate (5–6), and low (<5). This scoring approach, while not prescribed by CASP, aligns with practices described in a study advocating pragmatic adaptations of the tool to enhance its usability in qualitative evidence synthesis [36].

The MMAT is a critical appraisal tool designed to evaluate, among other things, the quality of mixed methods studies. It can be used to assess the quality of primary studies based on experimental or observational data. When appraising mixed-methods studies, the rationale for using a mixed-methods approach is first examined, followed by separate evaluations of the qualitative and quantitative components. In line with guidance from the MMAT developers, we did not calculate an overall numeric score, in order not to oversimplify nuanced methodological judgments [34]. Instead, we reported criterion-level ratings and synthesized findings narratively, highlighting recurring strengths. This approach allowed us to transparently interpret the methodological rigor of each study and consider its influence on the synthesis. Articles that scored between 11 and 15 were categorized as high quality; 7–10, moderate; and less than 7 were considered low.

### Data management and analysis

We conducted an inductive thematic analysis [37]. From the extracted data, we generated line-by-line codes related to our research questions. Codes were grouped into categories based on their relationships with each other and on conceptual coherence, organized to reflect broader levels of abstraction, assigned to the most fitting category, and refined through consensus among reviewers. Using NVivo 15, each article was read closely, and codes were highlighted and assigned to inductively created nodes. Related nodes were then organized into hierarchies, with parent nodes representing broader categories and child nodes capturing specific codes. Queries, including word frequency, text search, and matrix coding, were used to explore relationships across categories and identify recurring patterns. Coding was performed independently by two reviewers, compared using NVivo’s coding comparison query, and refined through consensus. This iterative process elevated categories into overarching themes, which formed the basis for the synthesis of definitions and attributes of health system resilience. For the attributes of a resilient health system, 28 categories emerged from the codes generated from the extracted data. Using a thematic framework, these categories were further categorized into themes, from which we derived our attributes of a resilient health system. We used Microsoft Excel to describe articles by country, study type, building block of the health system, and shock. We used QGIS to map the countries whose health systems were studied. The basemap for the map was sourced from the Africa Shapefiles dataset on openAFRICA (http://open.africa/dataset/africa-shapefiles), licensed under the Open Data Commons Open Database Licence (ODbL) and Creative Commons Attribution 4.0 International (CC BY 4.0).

## Results

### Description of included articles

**Fig 1:**
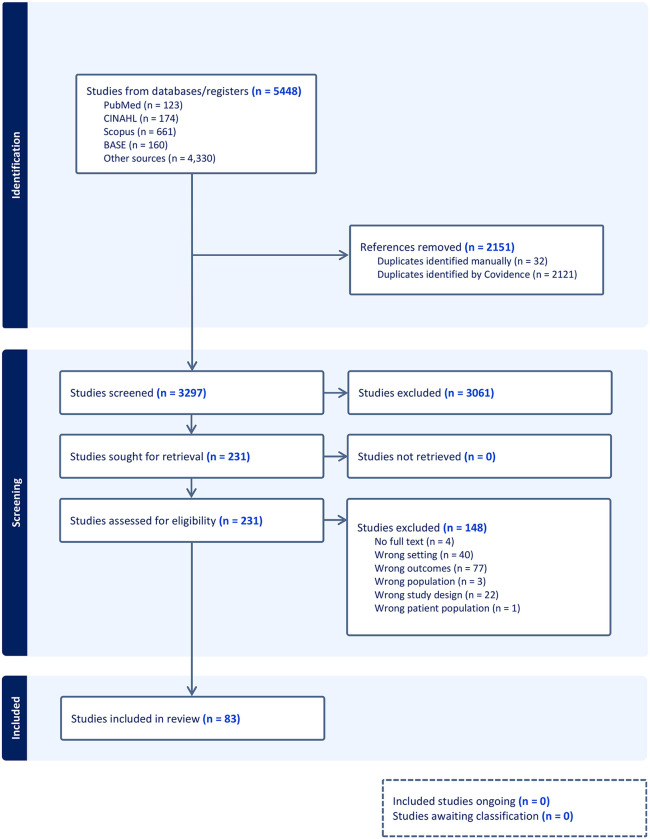
The PRISMA flow diagram.

The overall search yielded 5,448 records, 4,330 of which were from grey literature and forward citations. The rest were from the selected electronic databases, PubMed, CINAHL, Scopus and BASE. We removed 2,151 duplicate records and consequently screened 3,297 for relevance based on titles and abstracts. During screening, we excluded 3,061 articles for reasons including inappropriate populations or outcomes, studies conducted outside Africa, unsuitable study designs, publication types, and shocks other than infectious diseases. The title and abstract screening yielded 217 records for full-text review. During full-text screening, 133 articles were excluded for various reasons. We included 83 articles for review as shown in the PRISMA flow diagram in Fig 1.

**Fig 2:**
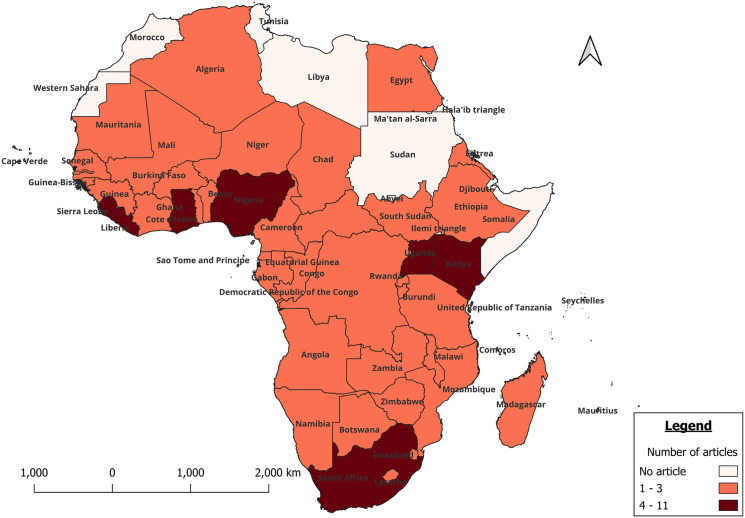
Countries represented in the included articles.

Almost three-quarters (60/83, 74.5%) of the included articles focused on COVID-19. This was followed by Ebola (16/83, 20%). Of the 54 African countries, 48 were represented in the included articles (Fig 2). Up to 78 of the 83 included articles (94.0%) studied service delivery as a health system building block, 64/83 (77.1%) addressed human resources for health; health information systems was the least discussed building block, featuring in only 18 of the 83 (27.5%) articles (Table 1).

**Table 1:** Characteristics and distribution of included articles.

S/No	Author, year of publication	Title of study	Publication type	Study design	Country of study	Definition of resilience	Attributes of resilience	Health system building block
1	Gebru et al., 2025 [38]	The crucial role the field epidemiology training program played in preparedness and response to the COVID-19 pandemic in Sierra Leone, January 2020 to August 2022	Research article	Qualitative research	Sierra Leone	The capacity for identification, investigation, response and communicating about public health threats	1. Coordination of preparedness activities 2. Point of entry screening 3. Investigation of COVID-19 cases 4. Strengthening contact tracing 5. Epidemiological and operational research	1. Service Delivery; 2. Health Workforce
2	Dhillon et al., 2025 [39]	We’re More Prepared than Before: Understanding the Strategies Used by a Non-governmental Organization During the COVID-19 Pandemic in Sub-Saharan Africa	Research article	Qualitative research	Uganda	The ability of groups and institutions to maintain system function when responding to significant disturbances such as the COVID-19 pandemic or other disasters	1. Adaptive capacity 2. Transformative capacity 3. Coping capacity	1. Leadership and Governance; 2. Service Delivery; 3. Health Workforce
3	Padraig Lyons et al., 2025 [40]	Building confidence in crises - the roles of Sierra Leonean religious leaders’ during the 2014–2016 Ebola outbreak	Research article	Qualitative research	Sierra Leone	Withstanding a crisis	1. Trust in non-political actors 2. Community support 3. Collaboration	1. Leadership and Governance; 2. Service Delivery
4	Tchetche, 2021 [41]	Social Perceptions Influencing the Fight Against Covid-19 in Cote D’Ivoire	Research article	Qualitative research	Ivory Coast	Control of epidemics	1. Organizational and monitoring framework for Anti-COVID 2. Community healthcare workers 3. Community involvement	1. Leadership and Governance; 2. Service Delivery; 3. Health Workforce
5	Eyles et al., 2015 [42]	Endurance, resistance and resilience in the South African health care system: case studies to demonstrate mechanisms of coping within a constrained system	Research article	Qualitative research	South Africa	The ability to respond positively to adversity and crises	1. Coping 2. Resistance	1. Service Delivery
6	Mohammed, 2021 [43]	Preparedness and response to covid-19 in Woreta Town, North West Ethiopia	Research article	Qualitative research	Ethiopia		1. Coordination 2. Risk communication and community engagement 3. Rapid response 4. Surveillance 5. Laboratory 6. Infection prevention and control 7. Case management 8. Continuity of essential services 9. Logistics, procurement and supply management 10. Humanitarian assistance	1. Leadership and Governance; 2. Service Delivery; 3. Health Workforce
7	Yigzaw et al., 2025 [44]	Turning challenges into opportunities: Lessons from Ethiopia’s COVID-19 response for strengthening health systems and health security	Research article	Qualitative research	Ethiopia	Capacity to absorb, adapt and transform when exposed to a shock such as a pandemic, natural disaster or armed conflict and still retain the same control over its structure and functions	1. Multi-sectoral and multi-stakeholder collaboration 2. Training and deployment of RRTs 3. Contact tracing, quarantine and isolation 4. Availability of quality data for surveillance 5. Surveillance at PoE 6. COVID-19 laboratory testing 7. Case management 8. Logistics and supply systems 9. Vaccination 10. Service integration	1. Leadership and Governance; 2. Service Delivery; 3. Health Workforce
8	Stephens et al., 2024 [45]	The potential risk components and prevention measures of the Ebola virus disease outbreak in Liberia: An in-depth interview with the health workers and stakeholders	Research article	Qualitative research	Liberia	Withstanding a shock	1. Surveillance 2. Increased awareness 3. Contact tracing 4. Good hygiene practices	1. Leadership and Governance; 2. Service Delivery; 3. Health Workforce
9	Posse et al., 2025 [46]	Lessons learnt in the response to COVID-19 in Mozambique: enabling readiness for the next pandemic	Research article	Qualitative research	Mozambique	Control of the COVID-19 pandemic	1. Use of data for decision-making 2. Monitoring rumors about vaccines 3. Mobilization of surge staff 4. Innovation, flexibility, and adaptability 5. Investments in the vaccine supply chain 6. Phased allocation of vaccines 7. Communication packages used by religious leaders 8. Sentinel surveillance of flu-like syndromes and AEFIs	1. Leadership and Governance; 2. Service Delivery; 3. Health System Financing; 4. Health Workforce; 5. Medical products, vaccines and technologies
10	Odhiambo Onyango et al., 2024 [47]	Availability of essential medicines during the COVID-19 pandemic: A qualitative study examining experiences and level of preparedness in Kenya	Research article	Qualitative research	Kenya	Availability and access to essential medicines during a crisis	1. Financing for essential medicines 2. Supply chain and procurement systems for essential medicines 3. Regulatory policies and manufacturing capacity of essential medicines 4. Human resource for health training 5. Health information system on essential medicines	1. Leadership and Governance; 2. Service Delivery; 3. Health Workforce; 4. Medical products, vaccines and technologies; 5. Health Information Systems
11	Niyigena et al., 2021 [48]	Rwanda’s community health workers at the front line: a mixed-method study on perceived needs and challenges for community-based healthcare delivery during COVID-19 pandemic	Research article	Qualitative research	Rwanda	Ability to respond to current and future crisis	1. Continuum of health service delivery 2. Increasing access to PPE 3. Investment in training and supervision of CHWs 4. Improvements in supply chain management; 5. Financial compensation for CHWs in the COVID-19 response	1. Leadership and Governance; 2. Service Delivery
12	Ninsiima et al., 2025 [49]	Strategies Utilized During Sudan Virus Disease Outbreak Response in Kampala City, Uganda, 2022âˆ’2023	Research article	Qualitative research	Uganda	Capacity to withstand an outbreak	1. Activation of Incident Management System 2. KCCA call and dispatch centre for alert management 3. Case investigation and contact tracing 4. Laboratory 5. Efficient fleet system for EMS 6. Support from Partners 7. Ring infection prevention and control 8. IPC scorecard assessments and mentorships 9. Application of different mass media interventions	1. Leadership and Governance; 2. Service Delivery
13	Namuhani et al., 2024 [50]	Leveraging community health workers for COVID-19 response in Democratic Republic of Congo, Nigeria, Senegal, and Uganda: roles, barriers, and facilitators	Research article	Qualitative research	1. Uganda 2. Nigeria 3. Democratic Republic of Congo 4. Senegal	Continuation of the provision of essential healthcare services while at the same time supporting response to the outbreak	1. Training 2. Development and dissemination of strategies, guidelines, and SOPs 3. Provision of mobile phones and tools 4. Provision of PPE 5. Community trust in CHWs 6. Additional allowances for community health workers 7. Community will 8. The availability of community structures such as community radios 9. The provision of travel permits during curfews	1. Leadership and Governance; 2. Service Delivery; 3. Health Workforce
14	Motsumi and Nemakonde, 2025 [51]	Coping with COVID-19 using traditional medicine: perspectives from Joe Morolong, Northern Cape	Research article	Qualitative research	South Africa	Responding to the COVID-19 pandemic	1. Knowledge of traditional medicine agents 2. Indigenous governance institutions in the community 3. Medical institutions used during the COVID-19 4. Indigenous knowledge of preparation and use of agents	1. Leadership and Governance; 2. Medical products, vaccines and technologies
15	Mitike et al., 2024 [52]	Providers’ experience on essential health services in primary healthcare units of Ethiopia during COVID-19: a qualitative study on impact and response	Research article	Qualitative research	Ethiopia	Continuation of provision of essential healthcare services	1. Health education in the community 2. Dedicated planning team 3. Tracking of patients in the community 4. New staff hires	1. Service Delivery; 2. Health Workforce
16	Kunfah et al., 2025 [53]	The impact of COVID-19 on health system resilience to the cerebrospinal meningitis outbreak in the Upper West Region of Ghana: a qualitative study	Research article	Qualitative research	Ghana	Effectively respond to multiple crises simultaneously and future health emergencies	1. Governance and financing 2. Health workforce 3. Health service delivery 4. Public health functions 5. Medical products and technology 6. Community engagement	1. Leadership and Governance; 2. Service Delivery; 3. Health System Financing; 4. Health Workforce; 5. Medical products, vaccines and technologies
17	Komasawa et al., 2024 [54]	Lessons for Strengthening a Resilient Health System from the View of Health Facilities During the COVID-19 Pandemic: A Qualitative Study	Research article	Qualitative research	Uganda	Delivering essential health services in urban cities in Uganda during a health emergency	1. Positive Change in the Drug Supply Chain 2. Vertical Health System with Other Sectors 3. Securing Transportation 4. Risk Communication	1. Leadership and Governance; 2. Service Delivery; 3. Health System Financing; 4. Health Workforce; 5. Medical products, vaccines and technologies
18	Kawuma et al., 2025 [55]	Resilience amidst challenges: Healthcare users’ experiences of access and utilisation of primary healthcare services during the COVID-19 pandemic in southwestern Uganda	Research article	Qualitative research	Uganda	Capacity to identify and handle disruptions, large or small, and invoke mechanisms for systems to “bounce back” and establish or re-establish a “new normal” situation	1. Knowledge and sensitization about COVID-19 2. Enforcing the COVID-19 SOPs 3. Operational modifications 4. Physical structural changes	1. Leadership and Governance; 2. Service Delivery; 3. Health Workforce
19	Kallon et al., 2023 [56]	From policy to practice: a qualitive study exploring the role of community health workers during the COVID-19 response in Sierra Leone	Research article	Qualitative research	Sierra Leone		1. Continuing routine health services during COVID-19 2. Maximise the use of telehealth consultations and e-health 3. Ensure safe interaction for CHWs 4. Use of PPE 5. Continued monitoring, reporting and supervision 6. Provide mental health support 7. Provide incentives for CHWs 8. Provide training for CHWs	1. Leadership and Governance; 2. Service Delivery; 3. Health Workforce
20	Kabwama et al., 2022 [57]	Private sector engagement in the COVID-19 response: experiences and lessons from the Democratic Republic of Congo, Nigeria, Senegal and Uganda	Research article	Qualitative research	1. Democratic Republic of Congo 2. Nigeria 3. Senegal 4. Uganda	Effective response to the COVID-19 outbreak	1. Private sector engagement in the COVID-19 response 2. Private sector partnership in the response 3. Treatment and management of COVID-19 4. Risk communication and health promotion 5. Supporting continuity of access to other health services	1. Leadership and Governance
21	Jephcott et al., 2025 [58]	Practical norms in emerging infectious disease control: lessons for transnational collaboration from a suspected newly-emerging zoonosis outbreak in Ghana	Research article	Qualitative research	Ghana		1. Acquisition of specialised testing 2. Coordination through visionary leadership 3. The engineering and administration of a system in flux	1. Leadership and Governance; 2. Service Delivery
22	Guha et al., 2025 [59]	Maintaining essential health services during COVID-19: cross-country lessons of health system resilience from Asia, Sub-Saharan Africa and Latin America		Qualitative research	Uganda Ghana	The capacity to anticipate, absorb, respond and recover from shocks, while maintaining core functions and serving the ongoing and acute care needs of communities	1. A whole-of-government response with multisectoral collaboration 2. Early, effective outbreak control and case management 3. Partnerships with the private sector 4. Academic-government partnerships 5. Existing financing mechanism(s) for emergency response and EHS 6. Expansion of health financing schemes 7. Government policies to prioritise maintenance of EHS 8. Service delivery adaptations and provision of care through alternative modalities 9. Rapid mobilisation and deployment of health workforce 10. Operational flexibility 11. Adaptation of existing health infrastructure 12. Mental health and psychosocial support 13. Robust community engagement efforts	1. Leadership and Governance; 2. Service Delivery; 3. Health Workforce
23	Dwomoh et al., 2023 [60]	COVID-19 outbreak control strategies and their impact on the provision of essential health services in Ghana: An exploratory- sequential study	Research article	Qualitative research	Ghana	Ability to control the COVID-19 outbreak and maintain access to safe and essential healthcare services	Controlling outbreak 1. Rapid response coordination 2. Adoption of pre-existing surveillance system 3. Implementation of testing strategies 4. Sustained multisectoral coordination 5. Public engagement Maintaining essential health services 1. Persuasion and incentivisation 2. Enablement and environment restructuring 3. Restriction	1. Service Delivery; 2. Health Workforce
24	Berhane et al., 2024 [61]	Lessons learned from Ethiopia’s preparedness and initial response to the Covid-19 pandemic	Research article	Qualitative research	Ethiopia		1. Coordination and multisectoral collaboration 2. Risk communication 3. Enhanced surveillance 4. Improved testing capacities 5. Infection prevention and control 6. Strong case management 7. Supplies and logistics	1. Leadership and Governance; 2. Service Delivery
25	Bello et al., 2024 [62]	Health systems challenges, mitigation strategies and adaptations to maintain essential health services during the COVID-19 pandemic: learnings from the six geopolitical regions in Nigeria	Research article	Qualitative research	Nigeria	Recovery in service volumes within three months of decline precipitated by the COVID-19 pandemic.	1. Leadership and governance 2. Financing 3. Service delivery 4. Human resources 5. Medicines and supplies	1. Leadership and Governance; 2. Service Delivery; 3. Health System Financing; 4. Health Workforce; 5. Medical products, vaccines and technologies
26	Balde et al., 2024 [63]	Responding to fluctuations in public and community trust and health seeking behaviour during the COVID-19 pandemic: a qualitative study of national decision-makers’ perspectives in Guinea and Sierra Leone	Research article	Qualitative research	Guinea and Sierra Leone		1. Community engagement 2. PPE distribution 3. Misinformation 4. Availability of community healthcare workers	1. Health Workforce
27	Aubourg et al., 2022 [64]	A Qualitative Evaluation of COVID-19 Preventative Response Activities in South Kivu, Democratic Republic of the Congo	Research article	Qualitative research	Democratic Republic of Congo		1. Community-level awareness 2. Political trust 3. Stakeholder collaboration 4. Investment in scientific research	1. Leadership and Governance; 2. Health Workforce
28	Agboh et al., 2024 [65]	Qualitative insights on emergency preparedness and response to Marburg Virus Disease in Ghana: The role of risk communication and community engagement	Research article	Qualitative research	Ghana		1. Community engagement and 2. Iinfodemic management 3. Coordinated and informal approach to risk communication 4. Stakeholder collaboration for risk communication	1. Service Delivery
29	Adongo et al., 2017 [66]	Health workers perceptions and attitude about Ghana’s preparedness towards preventing, containing, and managing EVD		Qualitative research	Ghana	Early detection and isolation of suspected cases through screening of international travellers	1. Training 2. Provision of PPE 3. Healthcare worker positive attitude	1. Service Delivery
30	Abu and Elliott, 2022 [67]	The critical need for WASH in emergency preparedness in health settings, the case of COVID-19 pandemic in Kisumu Kenya	Research article	Qualitative research	Kenya		1. Collaboration with local governments 2. Cultural sensitivity 3. Political will 4. Investment in emergencies “war chest”	1. Leadership and Governance; 2. Service Delivery
31	Karamagi et al., 2022 [22]	On the resilience of health systems: A methodological exploration across countries in the WHO African Region	Research article	Qualitative research	Health systems in the Africa Region of WHO	The capacity to prepare and effectively respond to crises; maintain core functions; and, informed by lessons learnt, reorganize if conditions require it	1. Legislation; 2. IHR coordination; 3. Zoonotic events; 4. Food safety; 5. Laboratory; 6. Surveillance; 7. Human resources; 8. Emergency framework; 9. Service provision; 10. Risk communication; 11. Points of entry; 12. Chemical events; 13. Radiation emergencies; 14. Awareness; 15. Diversity; 16. Self-regulation; 17. Mobilization; 18. Transformation	1. Leadership and Governance; 2. Service Delivery; 3. Health System Financing; 4. Health Workforce; 5. Health Information Systems
32	Ling et al., 2017 [68]	Beyond the crisis: did the Ebola epidemic improve resilience of Liberiaâ€™s health system?	Research article	Qualitative research	Liberia	Health system resilience has been defined as the capacity to prepare for and effectively respond to crises while maintaining core health system functions pre-, during, and post-crisis	1. Aware 2. Diverse 3. Self-regulating, 4. Integrated 5. Adaptive	1. Leadership and Governance; 2. Service Delivery; 3. Health System Financing
33	Haldane et al., 2021 [21]	Health systems resilience in managing the COVID-19 pandemic: lessons from 28 countries	Review article	Qualitative research	Liberia, Mozambique, Niger, Nigeria, Uganda	Health systems resilience literature stresses that efforts should focus not only on absorbing unforeseen shocks, but also on ensuring continuity in health improvement, sustaining gains in systems functioning and fostering people-centeredness, while delivering high-quality care	1. Community engagement 2. Testing 3. Contact tracing, 4. Non-pharmaceutical interventions, 5. Health information systems	1. Service Delivery; 2. Health Information Systems
34	Oga-Omenka et al., 2023 [69]	Tuberculosis service disruptions and adaptations during the first year of the COVID-19 pandemic in the private health sector of two urban settings in Nigeria: A mixed methods study	Research article	Cross sectional study	Nigeria	Continued provision of essential services	1. Integration of TB and COVID-19 testing; 2. Collaboration and coordination among the private sector and government entities 3. Active engagement of the private sector	1. Service Delivery; 2. Health System Financing
35	Iliyasu et al., 2023 [70]	˜We delivered at home out of fear: Maternity Care in Rural Nigeria During the COVID-19 Pandemic	Research article	Cross sectional study	Nigeria	Ability to withstand the outbreak while able to provide quality maternal health services	1. Community TBAs, 2. Adherence to preventive measures	1. Service Delivery
36	Sripad et al., 2022 [71]	Confirming and testing bonds of trust: A mixed methods study exploring community health workers’ experiences during the COVID-19 pandemic in Bangladesh, Haiti and Kenya	Research article	Cross sectional study	Kenya	Continued provision of healthcare services by CHW through a period of crisis	1. Trust 2. Availability of CHWs	1. Service Delivery; 2. Health Workforce
37	Hassem, et al., 2022 [72]	COVID-19: Contrasting experiences of South African physiotherapists based on patient exposure	Research article	Cross-sectional study	South Africa	The extent of a respondent’s ability to adapt well during significant stressful circumstances	1. Social support systems 2. Increased communication 3. Peer support from immediate colleagues, 4. Improved disaster management protocols 5. Improved PPE provision 6. Increased staffing. Access to professional psychological services 7. Implementation and enforcement of safety protocols	1. Health Workforce
38	Drevin et al., 2019 [73]	“For this one, let me take the risk”: why surgical staff continued to perform caesarean sections during the 2014 – 2016 Ebola epidemic in Sierra Leone	Research article	Qualitative research	Sierra Leone	A resilient health system may withstand shocks such as an EVD outbreak	1. Leadership 2. Solidarity 3. Adequate workforce, 4. Bottom-up decision making	1. Leadership and Governance; 2. Service Delivery; 3. Health Workforce
39	Itaye et al., 2023 [74]	Our interventions are still here to support communities during the pandemic: Resuming mass drug administration for neglected tropical diseases after COVID-19 implementation delays	Research article	Qualitative research	10 African countries	Ability to continue performing during and beyond global pandemics	1. Infection prevention and control 2. Community mobilization 3. Embracing technology and innovation, 4. Integration of programs	1. Leadership and Governance 2. Service Delivery
40	Ogira et al., 2022 [75]	Identifying the impact of COVID-19 on health systems and lessons for future emergency preparedness: A stakeholder analysis in Kenya	Research article	Cross sectional study	Kenya	Health system that is able to cater for needs from a pandemic while maintaining routine health services	1. Provision of emergency supplies 2. Strong surveillance & health information systems 3. Availability of adequate human resources 4. Adequacy of health infrastructure 5. Adequacy of financing 6. Coordination between the national and local governments	1. Leadership and Governance 2. Service Delivery; 3. Health System Financing 4. Health Workforce; 5. Medical products, vaccines and technologies 6. Health Information Systems
41	Ware et al., 2023 [76]	How Central Ugandan HIV Clinics Adapted During COVID-19 Lockdown Restrictions to Promote Continuous Access to Care: A Qualitative Analysis	Research article	Qualitative research	Uganda	The ability of a health system to adapt	1. Multi-month refills; 2. Facilitating visits to clinics closer to home 3. Delivering medication refills in the neighborhood	1. Leadership and Governance 2. Service Delivery
42	McKay et al., 2021 [77]	Safely resuming neglected tropical disease control activities during COVID-19: Perspectives from Nigeria and Guinea	Policy paper	Qualitative research	Guinea and Nigeria	Continued provision of NTD services despite crisis	1. Lesson learning, integration 2. Intersectoral collaboration 3. COVID-19 training, 4. Information sharing on risk reduction, 5. Community engagement 6. Embracing technology 7. Creative innovation, 8. Awareness and advocacy	1. Leadership and Governance 2. Service Delivery; 3. Health Workforce
43	McCollum et al., 2022 [78]	Qualitative study exploring lessons from Liberia and the UK for building a people-centred resilient health systems response to COVID-19	Research article	Qualitative research	Liberia	Improving resilience within health systems can build on existing strengths to enhance the readiness of health system actors to respond to crises while maintaining core functions.	1. Flexible pathways for medical supplies; 2. Priority list of EHS 3. Trust with local communities 4. Good communication 5. Recognition of staff; 6. Rapid resource flow 7. Agile tracking of health information 8. Effective partnerships 9. Advanced preparedness; 10. Adaptive governance and leadership 11. Effective coordination of response	1. Leadership and Governance 2. Service Delivery; 3. Health Workforce; 4. Health Information Systems
44	Ojielo et al., 2023 [79]	Analysis of the availability, effectiveness and equity of deployment of resources in the health system response to COVID-19 in Nigeria	R esearch article	Qualitative research	Nigeria	Effectively responding to the pandemic	Availability of effective and equitable countermeasures and interventions for response to COVID-19	1. Service Delivery; 2. Health Workforce; 3. Medical products, vaccines and technologies
45	Kabwama et al., 2024 [80]	How interventions to maintain services during the COVID-19 pandemic strengthened systems for delivery of maternal and child health services: a case-study of Wakiso District, Uganda	Research article	Qualitative research	Uganda	Health system resilience is a multi-dimensional concept that includes a system’s capacity to absorb, adapt, transform and improve during and after a crisis	1. Electronic health records 2. Medical devices 3. Teamwork, 4. Communication and coordination, 5. Supervision and management style 6. Work schedules 7. Social relationships 8. Health facility layout 9. Specific health policy 10. Information flow 11. Patient outcomes 12. Safety 13. Satisfaction 14. Care quality 15. Caregiver outcomes 16. Safety, stress and burnout Teamwork 17. Staff turnover	1. Leadership and Governance; 2. Service Delivery; 3. Health Workforce; 4. Health Information Systems
46	Curran et al., 2018 [81]	Systems, supplies, and staff: a mixed-methods study of health care workers’ experiences and health facility preparedness during a large national cholera outbreak, Kenya 2015	Research article	Mixed methods	Kenya	Health system successfully responding to a cholera outbreak	1. Multidisciplinary participation in outbreaks 2. Governance and coordination 3. Reporting and surveillance 4. Staffing 5. Utilization of healthcare services 6. Strong community health workforce 7. Strong health education,	1. Leadership and Governance; 2. Service Delivery; 3. Health Workforce; 4. Medical products, vaccines and technologies
47	Siekmans et al., 2017 [82]	Community-based health care is an essential component of a resilient health system: evidence from Ebola outbreak in Liberia	Research article	Qualitative research	Liberia	Building the resilience of communities during a crisis and ensuring prompt access to life-saving treatment for children at the community level.	1. Routine supervision of CHWs 2. Supply chain management 3. Referrals 4. Community engagement; 5. Training of CHWs 6. Provision of essential medicines to CHW	1. Service Delivery; 2. Health Workforce
48	Alhassan et al., 2023 [83]	Perceived impacts of COVID-19 responses on routine health service delivery in Liberia and UK: cross-country lessons for resilient health systems for equitable service delivery during pandemics	Research article	Qualitative research	Liberia	Assurance of delivery of essential health service	1. Use of technology; 2. Inclusive service provision 3. Cross-sectoral collaboration 4. Community involvement 5. Cultural sensitivity 6. Dignity and respect in countering misinformation; 7. Deployment of surge staff 8. Staff collaboration and teamwork;	1. Leadership and Governance; 2. Service Delivery; 3. Health Workforce; 4. Medical products, vaccines and technologies; 5. Health Information Systems
49	Saito et al., 2023 [23]	Enhancing community health system resilience: lessons learnt during the COVID-19 pandemic in Uganda through the qualitative inquiry of the COVID Task Force	Research article	Qualitative research	Uganda	Facilities providing quality care	1. Knowledge 2. Financial resources, 3. Communication, 4. Governance 5. Human, social, and physical capital	1. Leadership and Governance 2. Service Delivery; 3. Health System Financing; 4. Health Workforce
50	Raven et al., 2018 [84]	Health workers’ experiences of coping with the Ebola epidemic in Sierra Leone’s health system: a qualitative study	Research article	Qualitative research	Sierra Leone	The ability to absorb shocks and maintain services in the face of them	1. Strong links with the community; 2. Trust; 3. Community involvement; 4. Peer networks; 5. ICT integration Training, 6. Social media, 7. Workshops, 8. Financial support, Religion, 9. A sense of patriotism, Family support	1. Leadership and Governance; 2. Service Delivery; 3. Health Workforce
51	Lohmann et al., 2023 [85]	Stress and coping in the face of COVID-19: a qualitative inquiry into early pandemic experiences & psychological well-being of health workers in Burkina Faso, Senegal and The Gambia	Research article	Qualitative research	Burkina Faso, Senegal and The Gambia	Coping with crisis	1. Coping: precautions, Self-education 2. Stress management 3. Religion, 4. Social support, 5. Professionalism, 6. Faith 7. Learning, 8. Psychological preparation	1. Health Workforce
52	Yeboah et al., 2023 [86]	Maintaining essential health services during COVID-19 in Ghana: a qualitative study	Research article	Qualitative research	Ghana 148775		1. Psychosocial care by families 2. Home-based care; 3. Allocation of funds; 4. Triage stations; 5. Redistribution and rotation of staff; 6. Appointment scheduling 7. Provision of logistics; 8. Training; 9. Telemedicine; 10. Transportation support; 11. Incentives	1. Service Delivery; 2. Health Workforce
53	Schots et al., 2022 [87]	The impact of the COVID-19 pandemic on healthcare access and utilization in South Sudan: a cross-sectional mixed methods study	Research article	Cross-sectional study	South Sudan		1. Community knowledge and awareness; 2. Perceived need for care, 3. Perceived impact to community livelihoods 4. Community response to COVID-19	1. Service Delivery
54	Simen-Kapeu et al., 2021 [88]	Strengthening the community health program in Liberia: Lessons learned from a health system approach to inform program design and better prepare for future shocks	Research article	Qualitative research	Liberia		1. Communication 2. Engagement 3. Supervision/quality assurance	1. Service Delivery; 2. Health Workforce
55	Leung et al., 2022 [89]	Perceptions and experiences of maternity care workers during COVID-19 pandemic in Lagos State, Nigeria; a qualitative study	Research article	Qualitative research	Nigeria	Maintenance of maternal health services during COVID-19	1. Motivation 2. Adaptation, 3. Faith, 4. Community support, 5. Training, 6. Preparedness, 7. Collaboration 8. Adaptation over time, 9. Health system improvements	1. Service Delivery; 2. Health Workforce
56	Miller et al., 2018 [90]	Community health workers during the Ebola outbreak in Guinea, Liberia, and Sierra Leone	Research article	Systematic review	Guinea, Sierra Leone and Liberia	The capacity of health actors, institutions, and populations to prepare for and effectively respond to crises; maintain core functions when a crisis hits; and, informed by lessons learned during the crisis, reorganize if conditions require it	1. Motivation, 2. Community health workforce (TBAs and CHC members), 3. Referral compliance, 4. Service delivery	1. Service Delivery; 2. Health Workforce
57	Neill et al., 2023 [91]	Everyday capabilities were a path to resilience during COVID-19: a case study of five countries	Research article	Qualitative research	Ethiopia	The ability of the health system to prepare and respond to crises while maintaining basic services, adapting and learning from external shock	1. Interdependency of response 2. Lessons learnt 3. Public health capacity; 4. Primary health care; 5. Data and surveillance capacity 6. Leadership	1. Leadership and Governance; 2. Service Delivery; 3. Health Workforce
58	Amarakoon 2023 [92]	Exploring health information system resilience during COVID-19 pandemic: case studies from Norway, Sri Lanka & Rwanda	Research article	Other: Case study	Rwanda	Capacity to maintain high quality healthcare services by adapting to changes and challenges at different levels	1. Bouncing back and bouncing forward 2. Agility 3. Socio-technical determinants 4. Existing local capacity; 5. Networking & collaboration; 6. Technology/platform; 7. Enabling antecedent conditions	1. Health Information Systems
59	Kashiya, et al., 2023 [93]	Multilevel Governance and Control of the COVID-19 Pandemic in the Democratic Republic of Congo: Learning from the Four First Waves	Research article	Qualitative research	Democratic Republic of Congo		1. Lesson learning, 2. Well-streamlined structures	1. Leadership and Governance
60	Barker et al., 2020 [94]	Community engagement for health system resilience: evidence from Liberia’s Ebola epidemic	Research article	Qualitative research	Liberia	The capacity of health actors, institutions, and populations to prepare for and effectively respond to crises; to maintain core functions when a crisis hits; and, informed by lessons learned during the crisis, to reorganize if conditions require it	1. Increased trust, 2. Improved communication, 3. Solving problems in partnership with communities 4. Decentralized decision-making 5. Political support	1. Leadership and Governance; 2. Service Delivery
61	Accoe et al., [95]	Conditions for health system resilience in the response to the COVID-19 pandemic in Mauritania	Research article	Qualitative research	Mauritania	The capacity to manage and anticipate uncertainties, coordinate with key players and respond to community needs	1. Information sharing 2. Ability to anticipate 3. Maintaining a system-wide vision 4. Continuity of services 5. Proactive leadership 6. Partner support 7. Leadership structure 8. Down-top and top-down decision making 9. Trust 10. Clear communication 11. Involvement of CSOs	1. Leadership and Governance; 2. Service Delivery; 3. Health Workforce
62	Ridde et al., 2023 [96]	Hospital Resilience to the COVID-19 Pandemic in Five Countries: A Multiple Case Study	Editorial	Qualitative research	Mali	The capacities of a hospital facing shocks, stress or chronic destabilizing tensions (unexpected or anticipated, sudden or subtle, internal or external to the system), to absorb, adapt and/or transform in order to maintain and/or improve universal access to comprehensive, relevant, and high-quality health care without causing patients to fall into poverty.		1. Service Delivery
63	Gilson et al., 2017 [97]	Everyday resilience in district health systems: emerging insights from the front lines in Kenya and South Africa	Research article	Qualitative research	Kenya, and South Africa	The maintenance of positive adjustment under challenging conditions such that the organization emerges from those conditions strengthened and more resourceful	1. Governance, 2. Decentralization, 3. Community buy-in, 4. Financing, 5. Collaborative decision making	1. Leadership and Governance; 2. Service Delivery; 3. Health System Financing; 4. Health Workforce
64	Gizelis et al., 2017 [98]	Maternal Health Care in the Time of Ebola: A Mixed-Method Exploration of the Impact of the Epidemic on Delivery Services in Monrovia	Research article	Qualitative research	Liberia	The ability of the health system to continue providing services (maternal healthcare) during a crisis	1. Private sector involvement 2. Community health workforce 3. Social cohesion, 4. Trust	
65	Alonge et al., 2019 [99]	Understanding the role of community resilience in addressing the Ebola virus disease epidemic in Liberia: a qualitative study (community resilience in Liberia)	Research article	Qualitative research	Liberia	A set of attributes or dynamic social processes that indicate the adaptive (ability to continue with normal function), absorptive (magnitude of stress that can be withstood while maintaining normal function) and restorative capacities (ability to get back to normal function) in response to a health shock	1. A sense of strong community cohesion 2. Addressing misinformation	1. Leadership and Governance; 2. Service Delivery
66	Bang et al., 2020 [100]	Gauging Cameroon’s Resilience to the COVID-19 Pandemic: Implications for Enduring a Novel Health Crisis	Research article	Qualitative research	Cameroon	The ability of a system, community or society exposed to hazards to resist, absorb, accommodate to and recover from the effects of a hazard in a timely and efficient manner, including through the preservation and restoration of its essential basic structures and function	1. Communication, 2. Decentralization of response 3. Political commitment, 4. Investment in health infrastructure	1. Leadership and Governance; 2. Service Delivery; 3. Medical products, vaccines and technologies
67	Akinyemi et al., 2021 [101]	Qualitative exploration of health system response to COVID-19 pandemic applying the WHO health systems framework: Case study of a Nigerian state	Research article	Qualitative research	Nigeria		1. Well-functioning health system	2. Leadership and Governance; 3. Service Delivery; 4. Health System Financing; 5. Health Workforce; 6. Medical products, vaccines and technologies; 7. Health Information Systems
68	Tirivangani et al., 2021 [102]	Impact of COVID-19 pandemic on pharmaceutical systems and supply chain: a phenomenological study	Research article	Qualitative research	Namibia		1. Pharmacists as counselors 2. Supply chain 3. Effective patient-pharmacist interaction, 4. Strong preparedness and planning 5. Local manufacturing,	1. Medical products, vaccines and technologies
69	Chengo et al., 2022 [103]	A Situation Assessment of Community Health Workers’ Preparedness in Supporting Health System Response to COVID-19 in Kenya, Senegal, and Uganda	Research article	Cross-sectional study	Kenya, Uganda and Senegal	The ability of the health system to respond to the COVID-19 outbreak	1. Prepared and motivated CHWs; 2. Trained CHWs; 3. Equipping CHWs with PPE;	1. Service Delivery; 2. Health Workforce
70	Duby et al., 2022 [104]	Adaptation and Resilience: Lessons Learned From Implementing a Combination Health and Education Intervention for Adolescent Girls and Young Women in South Africa During the COVID-19 Pandemic	Research article	Other: Process evaluation	South Africa	Health systems ensure continuous SRH service provision to AGYW in times of pandemics and other crises	1. Provision of incentives 2. Reminders through WhatsApp groups and other strategies 3. Offering Door-to-Door Services:	1. Service Delivery
71	Ho et al., 2022 [105]	Health system resilience during COVID-19: Understanding SRH service adaptation in North Kivu	Research article	Cross sectional study	Democratic Republic of Congo	Capacity to continue providing essential healthcare services	1. Setting specific appointments to patients 2. Telephone communication, 3. Reducing group counselling to 5 people	1. Leadership and Governance; 2. Service Delivery; 3. Health Workforce
72	Kabwama et al., 2022 [106]	Interventions for Maintenance of Essential Health Service Delivery during the COVID-19 Response in Uganda, between March 2020 and April 2021	Research article	Qualitative research	Uganda	Resilient health systems involve a complex combination of interdependent interactions of health actors, institutions and populations, with the wherewithal to resist, prepare for and effectively control public health emergencies, maintain core functions during the emergencies and learn from them to transform and improve the system where necessary	1. Leadership and governance 2. Establishment of national committee on continuity of EHS 3. Risk communication and health promotion 4. Private sector engagement 5. Provision of IPC commodities 6. Review of supply plans, and emergency orders 7. Inter-warehouse transfers of essential products	1. Leadership and Governance; 2. Service Delivery; 3. Health System Financing; 4. Health Workforce; 5. Medical products, vaccines and technologies; 6. Health Information Systems
							8. Supplementary funding from government and international agencies 9. Continuation of regular funding by government 10. Development of guidelines and protocols for health worker safety, 11. Utilization of e-platforms to support supervision, training 12. Telemedicine, redeployment and recruitment of health workers 13. Provision of financial and non-financial incentives 14. Engaging community health workers 15. Designation of facilities for COVID-19 treatment, promotion of health self-management, 16. Provision of services in community settings, 17. Dispensing medicines for multiple months, 18. Leveraging patient networks to deliver medicines 19. Provision of data reporting tools	
73	Okyere et al., 2022 [107]	Covid-19 Front-Liners: Experiences of Palliative Care Providers in a Tertiary Hospital	Research article	Qualitative research	Ghana	Continued provision of quality palliative care services	1. Teleconsultation; 2. Adopting shift system 3. Reducing number of appointments; 4. Adoption of IPC strategies	1. Service Delivery; 2. Health Workforce
74	Jones-Konneh, 2023 [108]	Impact of health systems reform on COVID-19 control in Sierra Leone: a case study	Research article	Qualitative case study	Sierra Leone		1. Interdisciplinary approach, 2. IPC training, 3. Community awareness, 4. Surveillance and reporting, 5. Resource mobilization, 6. Collaboration with partners and donors, 7. Motivation of healthcare workers, 8. Testing and treatment, 9. Application of lessons learnt to strengthen health systems	1. Leadership and Governance; 2. Service Delivery
75	Komasawa, et al., 2023 [109]	Impact of Hospital Closure on Patients with Communicable and Non-Communicable Diseases During the COVID-19 Pandemic in Uganda: A Cross-Sectional and Mixed-Methods Study	Research article	Cross sectional study	Uganda	Uninterrupted delivery of healthcare services during a crisis	1. Access to healthcare services, 2. Healthcare expenditure, 3. Alternative healthcare, 4. Fear of stigma, 5. Functional outreach services	1. Service Delivery; 2. Health Workforce
76	Makwara et al., 2023 [110]	Exploring healthcare system adaptive techniques and challenges in caring for people living with HIV and AIDS during the COVID-19 lockdown period in Harare, Zimbabwe	Research article	Qualitative research	Zimbabwe	Health system resilience refers to health systems’ ability and capacity to absorb, effectively respond, and adapt to shocks and structural changes while sustaining day-to-day operations.	1. Standardized protocol, 2. COVID-19 preventive initiatives, 3. Communication, 4. Support groups for PLWHA	1. Leadership and Governance; 2. Service Delivery; 3. Health Workforce
77	Mohulatsi, 2023 [111]	The Experiences of Expectant and New Mothers in Accessing Maternal Healthcare Services during the COVID-19 Pandemic in Mmabatho, North-West, South Africa	Research article	Qualitative research	South Africa	Access of healthcare during COVID-19	1. Attitudes, 2. Social norms, 3. Perceived control, 4. Availability of social support, 5. Financial resources	1. Service Delivery
78	Neill et al., 2023 [112]	What made primary health care resilient against COVID-19? A mixed-methods positive deviance study in Nigeria	Research article	Mixed methods	Nigeria	Maintenance and recovery of PHC service volumes	1. Local government support for the response; 2. Dialogue with communities 3. Increasing the frequency of community engagement and outreach activities 4. Home visits; 6. Human resource reallocations 7. Surge capacity	1. Leadership and Governance; 2. Service Delivery; 3. Health System Financing; 4. Health Workforce
79	Reid et al., 2023 [113]	Tackling the first COVID-19 wave at the Cape Town Hospital of Hope: Why was it such a positive experience for staff?	Project report	Cross sectional study	South Africa	Systems that can better prevent, prepare for, and respond to future shocks, while maintaining essential health services	1. Leadership 2. Resources 3. Rapid decisions; 4. Uncertainty and fear 5. Person-centeredness; 6. Unity of purpose; 7. Teamwork and communication; 8. Staff support and safely; 9. Responsiveness and flexibility; 10. High job satisfaction, 11. Professional development; 12. Meaning 13. Service delivery	1. Leadership and Governance; 2. Service Delivery; 3. Health System Financing; 4. Health Workforce; 5. Medical products, vaccines and technologies; 6. Health Information Systems
80	Yeoh et al., 2023 [114]	Global health system resilience during encounters with stressors – Lessons learnt from cancer services during the COVID-19 pandemic	Research article	Qualitative research	Zambia and Egypt	The need to improve the functioning of health systems not only to meet emerging health needs caused by unforeseen shocks, but also to provide effective care for routine health needs	1. Improving the organization of health systems and pandemic preparedness 2. Building trust in governance and leadership 3. Identifying health system gaps for preparedness, readiness and response	1. Leadership and Governance; 2. Service Delivery; 3. Health System Financing; 4. Health Workforce; 5. Medical products, vaccines and technologies; 6. Health Information Systems
81	Guevart et al., 2008 [115]	Supervision formative: l’expérience des unités de traitement du choléra au cours de l’épidémie de Douala, Cameroun	Research article	Retrospective descriptive study	Cameroon	Provision of quality services during a crisis (the cholera outbreak)	1. Mentorship and coaching 2. Strengthened IPC measures 3. Documentation of processes 4. Development of protocols	1. Service delivery 2. Leadership and governance
82	Coulibaly et al., 2021 [116]	La résilience de l’hôpital du Mali face à la COVID-19 dans un contexte de pénuries	Research article	Cross-sectional study	Mali	It is a way of reacting to chronic stress in a way that transforms the way the institution operates	1. Mobilization of human resources 2. Financial resources 3. Infrastructure	1. Human resources for health 2. Health financing
83	Maltais et al., 2022 [117]	Comment la résilience post-Ebola en Guinée contribue à la gestion de la COVID-19?	Research article	Cross-sectional study	Guinea	The ability of a person or system to recover from a shock and remain relatively stable in a turbulent environment	1. Consciousness 2. Diversity 3. Awareness 4. Self-regulation 5. Adaptability	1. Service delivery 2. Leadership and governance

### Capacities generated from themes

Seventy-one articles (85.5%) discussed the description or definition of health system resilience. A critical review of the articles identified four main themes for resilience. These themes include preparedness, absorptive capacity, adaptive capacity, and recovery. We termed these themes capacities, which we then used to define the resilience of a health system. Separately, we identified seven attributes of a resilient health system.

### Capacities of health system resilience

Below, we describe the capacities that define health system resilience to infectious disease shocks in Africa.

#### Preparedness.

Preparedness of a health system strengthens its capacity to prevent public health crises, anticipate public health threats, and take the necessary steps to respond effectively. Studies conducted in Ghana, Uganda, Mauritania and Mali described health system resilience in their capacity to anticipate a crisis and act strategically to effectively counter it [59,95,96]. One study in South Africa, described the resilience of health systems in regard to its capacity “to take transformative action to build stronger, more resilient health systems that can better prevent, prepare for, and respond to future shocks [113]. Another study described early detection of disease as one of the interventions in preparedness [66].

#### Absorptive capacity.

The ability of a health system to continue providing essential health care services while responding to a shock is its absorptive capacity. The resilience of a health system refers to its capacity to maintain continuous healthcare services during and after a crisis [109]. This capacity for effective response reflects the health system’s ability to withstand shocks or manage crises it encounters [17,21,85].

#### Adaptive capacity.

The adaptive capacity of a health system includes its ability to anticipate challenges and put in place appropriate mechanisms to respond to them effectively [75,95]. Health systems, while they respond to emergencies, should have the ability to provide basic, as well as emerging, health needs during the response period [114]. They should be able to maintain their core functions, as well as hold on to a system-wide vision during a crisis [90,107,118]. Where physical access is not possible, as in lockdowns, there should be innovative ways of ensuring that essential healthcare services are available to all in need [111]. Health systems should be able to evolve from their pre-crisis state to develop structures that can withstand future events or put in place innovative measures to ensure that they fight back against the crisis while at the same time maintaining their core functions [92,110].

#### Recovery.

Health systems that can bounce back after a crisis and continue providing similar quality of care during, as before a crisis, is recovery [17,72,95,110]. One study in Nigeria described resilience with regard to the recovery in service within three weeks of the end of the crisis [62].

### Attributes of resilience

Below are the categories that we considered the attributes of a resilient health system.

#### Community engagement and involvement.

Community involvement in service delivery empowers residents to protect their own health [17]. Communities can participate in designing primary healthcare programs, raising awareness about prevention, and assessing resilience initiatives [82,83,87]. During emergencies, they play a key role in risk communication, guiding response teams, contact tracing, and reporting cases [71,77,81]. Such participation ensures culturally sensitive responses and fosters trust between health systems and communities [84,94].

#### Leadership and governance.

Leadership and governance are essential for policy development, enforcing strategic guidelines, and establishing regulatory mechanisms [17,22]. They ensure the availability of protocols and emergency frameworks that guide resilient operations [106,118], and coordinate alignment among stakeholders, including the private sector, international partners, health workers, and communities [74,78,81,118].

In Tanzania, government-private sector cooperation, as a leadership function, strengthened the resilience of pharmaceutical supply chains [17]. During outbreaks, such partnerships create multidisciplinary response teams, mobilize resources, and leverage expertise from governments, civil society, and community-based organizations [69,81,83,95,98].

Financing underpins resilience, but health services often face underfunding, especially at the community level where workers and programs remain unsupported [23,75,84,101,111]. In Uganda, filling gaps through government and partner support was recommended [106].

Decentralization enhances subnational systems by giving authority over resource distribution, service delivery, and outbreak response [94,100]. This bottom-up approach promotes efficient decision-making and close supervision, as demonstrated in Cameroon where decentralized responses improved resilience during both cholera and COVID-19 outbreaks [73,100,115].

#### Learning and adaptation.

Adaptability is crucial for strengthening a health system’s resilience [22]. It allows systems to respond to crises while maintaining essential functions [74,78]. Adaptive systems are flexible, capable of balancing external demands with quick internal reorganization as with Rwanda, with their health information systems, and Mali where a hospital put in place “multiple adaptation strategies that have resulted in mobilizing human resources to provide care to COVID-19 patients, creating infrastructure for the hospitalization of these patients, mobilizing financial resources to allow the start of the activities of the COVID-19 unit” [92,116]. Further, integrating programs promotes interdependence and efficient resource use [74,77,122]. Learning from past activities further enhances adaptive and transformative capacity [77,85,108,117].

Preparedness involves deliberate precautionary actions taken before a threat, such as training, developing protocols, and stockpiling measures [78]. In Namibia, preparedness and planning helped sustain pharmaceutical and supply chains during COVID-19 [102].

#### Health service delivery.

Access to quality healthcare is fundamental to system resilience [72,82,96]. Emergencies such as lockdowns and travel bans limit access, but innovative approaches are needed to maintain service continuity [76,106]. Primary healthcare (PHC), essential to universal health coverage, requires sufficient funding and workforce investment to uphold resilience [112]. Resilient systems stay responsive, timely, integrated, and inclusive [83,113]. With regards to infrastructure, physical facilities, equipment, and essential supplies, COVID-19 highlighted the importance of timely access to protective equipment, diagnostics, and medical technologies [17,23,75,100,101,106].

Health information systems enhance coordination and efficiency through effective data sharing [72,75,77,78,118,119]. Human resources for health are another cornerstone. Emergencies often divert staff from essential services, requiring innovative mechanisms such as surge rosters, redistribution, or reallocation of staff [83,86,112]. Capacity-building enhances preparedness, while teamwork fosters efficiency and solidarity [89,100,103,113,118]. Motivation through adequate incentives is equally vital [89,103,108].

#### Surveillance and laboratory.

Quality data supports effective surveillance, which is vital for the early detection of public health threats and guiding prompt responses [22,75,81,112]. Keeping laboratories functional during both normal periods and emergencies boosts resilience. Robust laboratory networks and field deployments have proven essential in mounting successful responses to Ebola outbreaks [22,120].

#### Innovation.

During the Ebola outbreak in Sierra Leone and the COVID-19 pandemic, technology and innovation proved vital for optimizing programs, planning, and data collection [74]. In emergencies, both technology-based and non-technology solutions are essential. Telemedicine and e-platforms help reduce facility crowding, maintain access during lockdowns, and support remote supervision [86,106,107]. Non-technological innovations include multi-month drug refills, using patient networks for medicine distribution, and organizing community clinics [76,106].

#### Health education and promotion.

Risk communication and health promotion enhanced resilience during COVID-19 in Uganda and among healthcare workers in South Africa [72,94,106]. Clear communication and strong health education empower communities with knowledge and awareness, enabling informed choices that support prevention [81,87].

### Quality assessment

Of the 62 qualitative articles included, 49 (79.0%) were of high quality, 7 (11.3%) of moderate quality, and the remainder of low quality. Up to 51/62 articles (82.3%) yielded valid results, as evidenced by responses to questions 1–6 of the assessment tool. Additionally, more than 86% of the studies reported considering ethical issues, employing sufficiently rigorous data analysis, and providing clear statements of findings. Based on the local application of these findings, over 90% of the articles were deemed relevant (Table 2).

**Table 2:** Assessment of quality of qualitative articles using CASP.

Author, year of publication	Title	Was there a clear statement of the aims of the research?	Is a qualitative methodology appropriate?	Was the research design appropriate to the aims of the research?	Was the recruitment strategy appropriate to the aims of the research?	Were the data collected in a way that addressed the research issue?	Has the relationship between researcher and participants been adequately considered?	Have ethical issues been taken into consideration?	Was the data analysis sufficiently rigorous?	Is there a clear statement of findings?	How valuable is the research?	CASP score
Gebru et al., 2025 [38]	The crucial role the field epidemiology training program played in preparedness and response to the COVID-19 pandemic in Sierra Leone, January 2020 to August 2022	1	1	1	1	1	-1	-1	1	1	1	6/10
Dhillon et al., 2025 [39]	We’re More Prepared than Before: Understanding the Strategies Used by a Non-governmental Organization During the COVID-19 Pandemic in Sub-Saharan Africa	-1	1	1	1	1	-1	1	1	1	1	6/10
Lyons et al., 2025 [40]	Building confidence in crises - the roles of Sierra Leonean religious leaders’ during the 2014–2016 Ebola outbreak	1	1	1	1	1	-1	1	1	1	1	8/10
Eyles et al., 2015 [42]	Endurance, resistance and resilience in the South African health care system: case studies to demonstrate mechanisms of coping within a constrained system	1	1	1	1	1	-1	1	1	1	1	8/10
Mohammed, 2021 [43]	Preparedness and response to covid-19 in Woreta Town, North West Ethiopia.	1	1	1	1	1	-1	1	1	1	1	8/10
Stephens et al., 2024 [45]	The potential risk components and prevention measures of the Ebola virus disease outbreak in Liberia: An in-depth interview with the health workers and stakeholders	1	1	1	1	1	-1	1	1	1	1	8/10
Posse et al., 2025 [46]	Lessons learnt in the response to COVID-19 in Mozambique: enabling readiness for the next pandemic	1	1	1	1	1	-1	1	1	1	1	8/10
Onyango et al., 2024 [47]	Availability of essential medicines during the COVID-19 pandemic: A qualitative study examining experiences and level of preparedness in Kenya	1	1	1	1	1	-1	1	1	1	1	8/10
Ninsiima et al., 2025 [49]	Strategies Utilized During Sudan Virus Disease Outbreak Response in Kampala City, Uganda, 2022–2023	1	1	1	1	1	-1	1	1	1	1	8/10
Namuhani et al., 2024 [50]	Leveraging community health workers for COVID-19 response in Democratic Republic of Congo, Nigeria, Senegal, and Uganda: roles, barriers, and facilitators	1	1	1	1	1	1	1	1	1	1	10/10
Motsumi and Nemakonde, 2025 [51]	Coping with COVID-19 using traditional medicine: perspectives from Joe Morolong, Northern Cape	1	1	1	1	1	-1	1	1	1	1	8/10
Mitike et al., 2024 [52]	Providers’ experience on essential health services in primary healthcare units of Ethiopia during COVID-19: A qualitative study on impact and response	1	1	1	1	1	-1	1	1	1	1	8/10
Kunfah et al., 2025 [53]	The impact of COVID-19 on health system resilience to the cerebrospinal meningitis outbreak in the Upper West Region of Ghana: a qualitative study	1	1	1	1	1	-1	1	1	1	1	8/10
Komasawa et al., 2024 [54]	Lessons for Strengthening a Resilient Health System from the View of Health Facilities During the COVID-19 Pandemic: A Qualitative Study	1	1	1	1	1	-1	1	1	1	1	8/10
Kawuma et al., 2025 [55]	“Resilience amidst challenges”: Healthcare users’ experiences of access and utilisation of primary healthcare services during the COVID-19 pandemic in southwestern Uganda	1	1	1	1	1	-1	1	1	1	1	8/10
Kallon et al., 2023 [56]	From policy to practice: a qualitive study exploring the role of community health workers during the COVID-19 response in Sierra Leone	1	1	1	1	1	-1	1	1	1	1	8/10
Kabwama et al., 2022 [57]	Private sector engagement in the COVID-19 response: experiences and lessons from the Democratic Republic of Congo, Nigeria, Senegal and Uganda	1	1	1	1	1	1	1	1	1	1	10/10
Jephcott et al., 2025 [58]	Practical norms in emerging infectious disease control: Lessons for transnational collaboration from a suspected newly emerging zoonosis outbreak in Ghana	1	1	1	1	1	-1	1	-1	-1	1	4/10
Guha et al., 2025 [59]	Maintaining essential health services during COVID-19: cross-country lessons of health system resilience from Asia, Sub-Saharan Africa and Latin America	1	1	1	1	1	-1	1	1	1	1	8/10
Dwomoh et al., 2023 [60]	COVID-19 outbreak control strategies and their impact on the provision of essential health services in Ghana: An exploratory sequential study	-1	1	1	1	1	0	1	1	1	1	7/10
Berhane et al., 2024 [61]	Lessons learned from Ethiopia’s preparedness and intial response to the COVID-19 pandemic	1	1	1	1	1	-1	1	1	1	1	8/10
Bello et al., 2024 [62]	Health systems challenges, mitigation strategies and adaptations to maintain essential health services during the COVID-19 pandemic: learnings from the six geopolitical regions in Nigeria	1	1	1	1	1	-1	1	-1	1	1	6/10
Balde et al., 2024 [63]	Responding to fluctuations in public and community trust and health seeking behaviour during the COVID-19 pandemic: a qualitative study of national decision-makers’ perspectives in Guinea and Sierra Leone	1	1	1	1	1	-1	1	1	1	1	8/10
Aubourg et al., 2022 [64]	A Qualitative Evaluation of COVID-19 Preventative Response Activities in South Kivu, Democratic Republic of the Congo	1	1	1	1	1	0	1	1	1	1	9/10
Agboh et al., 2024 [65]	Qualitative insights on emergency preparedness and response to marburg virus disease in Ghana: The role of risk communication and community engagement	1	1	1	0	1	-1	1	1	1	1	7/10
Adongo et al., 2017 [66]	Health workers perceptions and attitude about Ghana’s preparedness towards preventing, containing, and managing Ebola Virus Disease	1	1	1	1	1	-1	1	1	1	1	8/10
Abu et al., 2022 [67]	The critical need for WASH in emergency preparedness in health settings, the case of COVID-19 pandemic in Kisumu Kenya	-1	1	1	1	1	-1	1	-1	1	1	4/10
Ling et al., 2017 [68]	Beyond the crisis: did the Ebola epidemic improve resilience of Liberia’s health system?	1	1	1	1	1	-1	1	1	1	1	8/10
Haldane et al., 2021 [121]	Health systems resilience in managing the COVID-19 pandemic: lessons from 28 countries	1	1	1	1	1	-1	0	1	1	1	7/10
Drevin et al., 2019 [73]	“For this one, let me take the risk”: why surgical staff continued to perform caesarean sections during the 2014–2016 Ebola epidemic in Sierra Leone.	1	1	1	1	0	1	1	1	1	1	9/10
Ogira et al., 2022 [75]	Identifying the impact of COVID-19 on health systems and lessons for future emergency preparedness: A stakeholder analysis in Kenya.	1	1	1	1	1	0	1	1	1	1	9/10
Ware et al., 2023 [76]	How Central Ugandan HIV Clinics Adapted During COVID-19 Lockdown Restrictions to Promote Continuous Access to Care: A Qualitative Analysis.	1	1	1	-1	-1	-1	1	1	1	0	3/10
McKay et al., 2021 [77]	Safely resuming neglected tropical disease control activities during COVID-19: Perspectives from Nigeria and Guinea.	1	1	1	1	1	0	1	1	-1	1	7/10
McCollum et al., 2022 [78]	Qualitative study exploring lessons from Liberia and the UK for building a people-centred resilient health systems response to COVID-19.	1	1	1	0	1	-1	1	1	1	1	7/10
Ojielo et al., 2024 [79]	Analysis of the availability, effectiveness and equity of deployment of resources in the health system response to COVID-19 in Nigeria.	1	1	1	0	1	0	1	1	1	1	8/10
Kabwama et al., 2024 [80]	How interventions to maintain services during the COVID-19 pandemic strengthened systems for delivery of maternal and child health services: a case-study of Wakiso District, Uganda.	1	1	1	1	1	-1	1	1	1	1	8/10
Siekmans et al., 2017 [82]	Community-based health care is an essential component of a resilient health system: evidence from Ebola outbreak in Liberia.	1	1	1	1	1	-1	1	1	1	1	8/10
Alhassan et al., 2023 [83]	Perceived impacts of COVID-19 responses on routine health service delivery in Liberia and UK: cross-country lessons for resilient health systems for equitable service delivery during pandemics.	1	1	1	1	1	-1	1	1	1	-1	6/10
Saito et al., 2023 [23]	Enhancing community health system resilience: lessons learnt during the COVID-19 pandemic in Uganda through the qualitative inquiry of the COVID Task Force.	1	1	1	1	1	1	0	1	1	1	9/10
Raven et al., 2018 [84]	Health workers’ experiences of coping with the Ebola epidemic in Sierra Leone’s health system: a qualitative study.	1	1	1	1	1	0	1	1	1	1	9/10
Lohmann et al., 2023 [85]	Stress and coping in the face of COVID-19: a qualitative inquiry into early pandemic experiences and psychological well-being of health workers in Burkina Faso, Senegal and The Gambia.	0	1	1	1	1	-1	1	1	0	1	6/10
Yeboah et al., 2024 [86]	Maintaining essential health services during COVID-19 in Ghana: a qualitative study.	1	1	1	1	1	0	1	1	1	1	9/10
Simen-Kapeu et al., 2021 [88]	Strengthening the community health program in Liberia: Lessons learned from a health system approach to inform program design and better prepare for future shocks.	1	1	1	1	1	-1	1	1	1	1	8/10
Leung et al., 2022 [89]	Perceptions and experiences of maternity care workers during COVID-19 pandemic in Lagos State, Nigeria; a qualitative study.	1	1	1	1	1	-1	1	1	1	1	8/10
Amarakoon et al., 2023 [92]	Exploring health information system resilience during COVID-19 pandemic: case studies from Norway, Sri Lanka & Rwanda.	-1	1	1	1	1	0	1	1	1	1	7/10
Kashiya et al., 2023 [93]	Multilevel Governance and Control of the COVID-19 Pandemic in the Democratic Republic of Congo: Learning from the Four First Waves.	1	1	1	1	1	-1	1	1	1	1	8/10
Barker et al., 2020 [94]	Community engagement for health system resilience: evidence from Liberia’s Ebola epidemic.	1	1	1	1	1	-1	1	1	1	1	8/10
Accoe et al., 2023 [95]	Conditions for health system resilience in the response to the COVID-19 pandemic in Mauritania.	1	1	1	1	1	-1	1	1	1	1	8/10
Gilson et al., 2017 [97]	Everyday resilience in district health systems: emerging insights from the front lines in Kenya and South Africa	1	1	1	0	1	0	1	1	1	1	8/10
Alonge et al., 2019 [99]	Understanding the role of community resilience in addressing the Ebola virus disease epidemic in Liberia: a qualitative study (community resilience in Liberia)	1	1	1	1	1	1	1	1	1	1	10/10
Akinyemi et al., 2021 [101]	Qualitative exploration of health system response to COVID-19 pandemic applying the WHO health systems framework: Case study of a Nigerian state	1	1	1	1	1	1	1	1	1	1	10/10
Tirivangani et al., 2021 [102]	Impact of COVID-19 pandemic on pharmaceutical systems and supply chain - a phenomenological study	1	1	1	1	1	-1	1	1	1	1	8/10
Duby et al., 2022 [104]	Adaptation and Resilience: Lessons Learned From Implementing a Combination Health and Education Intervention for Adolescent Girls and Young Women in South Africa During the COVID-19 Pandemic	1	1	1	0	1	-1	1	0	-1	1	4/10
Kabwama et al., 2022 [106]	Interventions for Maintenance of Essential Health Service Delivery during the COVID-19 Response in Uganda, between March 2020 and April 2021	1	1	1	1	1	-1	1	1	-1	1	6/10
Okyere et al., 2022 [107]	Covid-19 Front-Liners: Experiences of Palliative Care Providers in a Tertiary Hospital	1	1	1	1	1	0	1	1	1	1	9/10
Jones-Konneh et al., 2023 [108]	Impact of health systems reform on COVID-19 control in Sierra Leone: a case study	1	1	1	1	1	-1	1	1	1	1	8/10
Makwara et al., 2023 [110]	Exploring healthcare system adaptive techniques and challenges in caring for people living with HIV and AIDS during the COVID-19 lockdown period in Harare, Zimbabwe	1	1	1	1	0	-1	1	1	1	1	7/10
Mohulatsi et al., 2023 [111]	The Experiences of Expectant and New Mothers in Accessing Maternal Healthcare Services during the COVID-19 Pandemic in Mmabatho, North-West, South Africa	1	1	1	1	1	0	1	1	1	1	9/10
Yeoh et al., 2023 [114]	Global Health System Resilience during Encounters with Stressors - Lessons Learnt from Cancer Services during the COVID-19 Pandemic	1	1	1	0	0	-1	1	0	1	1	5/10
Guevart et al., 2008 [115]	Supervision formative: l’expérience des unités de traitement du choléra au cours de l’épidémie de Douala, Cameroun	1	1	1	1	1	0	0	1	1	1	8/10
Coulibaly et al., 2021 [116]	La résilience de l’hôpital du Mali face à la COVID-19 dans un contexte de pénuries	1	1	1	1	1	0	0	1	1	1	8/10
Maltais et al., 2022 [117]	Comment la résilience post-Ebola en Guinée contribue à la gestion de la COVID-19?	1	1	1	1	1	0	0	1	1	1	8/10

Among the 21 mixed-methods articles evaluated using the MMAT, all passed screening questions 1 and 2. Only one was of a high quality; ten (47.6%) were of moderate quality; while the rest were of low quality (Table 3). While no papers were discarded for their lack of quality, their weight in the synthesis was handled with caution.

**Table 3:** Assessment of quality of qualitative articles using MMAT.

Author name, year	Title	Screening		Qualitative					Quantitative descriptive					Mixed methods				
		S1	S2	1.1	1.2	1.3	1.4	1.5	4.1	4.2	4.3	4.4	4.5	5.1	5.2	5.3	5.4	5.5
Karamagi et al., 2022 [22]	On the resilience of health systems: A methodological exploration across countries in the WHO African Region	Yes	Yes	Yes	Yes	Yes	Yes	Yes	Yes	Yes	Yes	No	Yes	Yes	Yes	Yes	Yes	Yes
Tchetche, 2021 [41]	Social Perceptions Influencing the Fight Against Covid-19 in Cote D’Ivoire	Yes	Yes	Yes	Yes	Yes	Yes	Yes	No	Yes	Yes	Yes	Yes	Yes	Yes	Yes	Yes	Yes
Yigzaw et al., 2025 [44]	Turning challenges into opportunities: Lessons from Ethiopia’s COVID-19 response for strengthening health systems and health security	Yes	Yes	Yes	Yes	Yes	Yes	Yes	Yes	Yes	Yes	No	Yes	Yes	Yes	Yes	Yes	Yes
Niyigena et al., 2021 [48]	Rwanda’s community health workers at the front line: a mixed-method study on perceived needs and challenges for community-based healthcare delivery during COVID-19 pandemic	Yes	Yes	Yes	Yes	Yes	Yes	Yes	Yes	Yes	Yes	No	Yes	Yes	Yes	Yes	Yes	Yes
Itaye et al., 2023 [74]	Our interventions are still here to support communities during the pandemic: Resuming mass drug administration for neglected tropical diseases after COVID-19 implementation delays	Yes	Yes	Yes	Yes	Yes	Yes	Yes	Yes	Yes	Yes	No	Yes	Yes	Yes	Yes	Yes	Yes
Ridde et al., 2023 [96]	Hospital Resilience to the COVID-19 Pandemic in Five Countries: A Multiple Case Study	Yes	Yes	Yes	Yes	Yes	Yes	Yes	Yes	Yes	Yes	No	Yes	Yes	Yes	Yes	Yes	Yes
Oga-Omenka et al., 2023 [69]	Tuberculosis service disruptions and adaptations during the first year of the COVID-19 pandemic in the private health sector of two urban settings in Nigeria: A mixed methods study	Yes	Yes	No	No	Yes	Yes	No	No	Yes	No	No	No	Yes	No	No	Yes	No
Iliyasu et al., 2023 [70]	˜We delivered at home out of fear: Maternity Care in Rural Nigeria During the COVID-19 Pandemic	Yes	Yes	No	No	Yes	Yes	No	No	Yes	Yes	No	Yes	No	No	Yes	No	No
Sripad et al., 2022 [71]	Confirming and testing bonds of trust: A mixed methods study exploring community health workers’ experiences during the COVID-19 pandemic in Bangladesh, Haiti and Kenya	Yes	Yes	No	No	Yes	No	No	No	No	Yes	No	No	Yes	Yes	No	No	Yes
Hassem et al., 2022 [72]	COVID-19: Contrasting experiences of South African physiotherapists based on patient exposure	Yes	Yes	Yes	No	Yes	No	No	No	No	No	No	No	No	No	Yes	Yes	No
Curran et al., 2018 [81]	Systems, supplies, and staff: a mixed-methods study of health care workers’ experiences and health facility preparedness during a large national cholera outbreak, Kenya 2015	Yes	Yes	No	No	No	Yes	No	Yes	No	No	Yes	No	Yes	Yes	Yes	Yes	No
Schots et al., 2022 [87]	The impact of the COVID-19 pandemic on healthcare access and utilization in South Sudan: a cross-sectional mixed methods study	Yes	Yes	Yes	No	No	Yes	Yes	Yes	No	Yes	No	Yes	No	No	Yes	Yes	Yes
Miller et al., 2018 [90]	Community health workers during the Ebola outbreak in Guinea, Liberia, and Sierra Leone	Yes	Yes	Yes	Yes	No	No	Yes	No	No	No	No	Yes	Yes	Yes	Yes	Yes	No
Neill et al., 2023 [91]	Everyday capabilities were a path to resilience during COVID-19: a case study of five countries	Yes	Yes	Yes	No	Yes	No	Yes	No	Yes	Yes	Yes	No	Yes	Yes	No	Yes	No
Gizelis et al., 2017 [98]	Maternal Health Care in the Time of Ebola: A Mixed-Method Exploration of the Impact of the Epidemic on Delivery Services in Monrovia	Yes	Yes	Yes	No	No	No	Yes	Yes	Yes	No	Yes	Yes	Yes	Yes	No	No	No
Bang et al., 2020 [100]	Gauging Cameroon’s Resilience to the COVID-19 Pandemic: Implications for Enduring a Novel Health Crisis	Yes	Yes	Yes	Yes	No	Yes	Yes	No	No	Yes	No	Yes	No	No	Yes	Yes	No
Chengo et al., 2022 [103]	A Situation Assessment of Community Health Workers’ Preparedness in Supporting Health System Response to COVID-19 in Kenya, Senegal, and Uganda	Yes	Yes	No	No	Yes	No	No	No	Yes	Yes	Yes	Yes	No	Yes	Yes	No	No
Ho et al., 2022 [105]	Health system resilience during COVID-19: Understanding SRH service adaptation in North Kivu	Yes	Yes	No	Yes	No	No	Yes	No	No	No	Yes	No	No	Yes	No	Yes	No
Komasawa et al., 2023 [109]	Impact of Hospital Closure on Patients with Communicable and Non-Communicable Diseases During the COVID-19 Pandemic in Uganda: A Cross-Sectional and Mixed-Methods Study	Yes	Yes	No	No	No	No	No	No	Yes	Yes	No	Yes	No	No	No	No	No
Neill et al., 2023 [112]	What made primary health care resilient against COVID-19? A mixed-methods positive deviance study in Nigeria	Yes	Yes	Yes	No	Yes	Yes	Yes	No	Yes	No	No	No	No	No	Yes	Yes	Yes
Reid et al., 2023 [113]	Tackling the first COVID-19 wave at the Cape Town Hospital of Hope: Why was it such a positive experience for staff?	Yes	Yes	Yes	Yes	No	No	No	Yes	No	No	No	No	Yes	Yes	No	No	Yes

## Discussion

Our review found that the description of health system resilience to infectious disease outbreaks relates to the level of preparedness, its capacity to absorb an outbreak when it occurs, its ability to adapt and sustain quality healthcare services during a response, and its capacity to recover after the crisis. We interpret these findings to suggest that the definition of a health system resilient to infectious disease shocks is a well-prepared system capable of anticipating and responding to imminent infectious disease emergencies; able to withstand such emergencies without severe adverse effects; capable of adapting and maintaining the provision of essential services while enhancing emergency response; and capable of recovering promptly after the emergency to continue delivering critical services.

This description highlights four essential capacities that must be actively maintained to be considered a resilient health system: preparedness; absorptive capacity; adaptive capacity; and recovery. Preparedness is defined as the capacity to anticipate potential threats and implement appropriate precautionary measures, such as training, stockpiling, and the development of protocols [122]. It encompasses all the necessary steps taken by the health system to enhance its capacity to appropriately detect, report and respond to a public health crisis. Absorptive capacity is the ability to maintain essential services during a shock [123]. The absorptive capacity of a health system enables it to respond to a health crisis or disaster with minimal interruption to the provision of essential healthcare services. Adaptive capacity refers to flexibility and innovation in sustaining services under new conditions [124]. Finally, recovery is the ability to restore service delivery to pre-crisis levels within a reasonable timeframe [125]. Recovery is differentiated from transformation, which concerns the overhauling of a health system guided by lessons learned from responding to previous crises.

Previous definitions of health system resilience reference three capacities: absorptive, adaptive, and transformative [126]. The definition outlined in our study encompasses preparedness and recovery; however, it does not include the concept of transformation capacities. Studies have emphasized the significance of preparedness as an essential component of resilience [127]. Another article observed that the destruction of health systems caused by COVID-19 was attributable to inadequate preparations, and deficiencies in robust surveillance and testing capabilities [128]. Preparedness has been linked to the absorptive capacity, as well as the adaptive capacities of a health system [123]. However, we argue that, to the extent that the benefit of preparedness includes prevention efforts, beyond just absorbing a shock, it should be considered a separate component capacity.

Our definition references recovery, which denotes returning to the pre-crisis state [129]. This requires reconsideration towards developing transformative capacities, which is a function of learning from previous crises and constructing a system capable of better responding to future shocks [130,131]. According to our study, the absorptive capacity of a health system refers to its ability to withstand a crisis, develop effective coping strategies, and maintain uninterrupted services. This aligns with the definitions found in several articles [123,127].

The attributes of resilient systems include leadership and governance, community engagement, health service delivery, health education and promotion, surveillance and laboratory, innovations, and continuous learning and adaptation. Earlier studies identified five attributes of a resilient health system: aware, integrated, diverse, self-regulating, and adaptive [18]. According to the article, these five ttributes, also called dimensions, are defined as: aware: the ability to detect risks early and understand its own strengths and vulnerabilities; diverse: offers a broad range of services and delivery models to meet health needs; self-regulating: the system can isolate threats and maintain core functions during crises; integrated: coordinates across sectors and engages communities to build trust and accountability; and adaptive: the system can learn from crises and adjust as may be required. There are considerable overlaps in the attributes between the subjects of our study and those discussed in the article; however, we will elaborate on community engagement and health education and promotion, which do not appear to be sufficiently addressed in the five dimensions.

The dimension “integrated” relates to the engagement of community leaders; however, numerous countries in Africa have health system frameworks that are embedded within the community, necessitating not merely involvement but active participation [132]. Community healthcare workers engage in the provision of primary healthcare services and serve as more than just intermediaries between the health system and the community [90]. Recognizing the role of the community as a major stakeholder in the development of resilient health systems, there have been calls for community inclusion as a fundamental component of the health system, beyond the traditional six building blocks of the health system [133]. Specifically in West Africa and Uganda, community healthcare workers fulfilled essential roles in managing outbreaks and in early detection of threats to facilitate an effective response [106,134]. Regarding community engagement, health education and promotion empower community members to take responsibility for their own health. Community healthcare workers and a community empowered with information on health are essential to the achievement of Universal Health Coverage, which resilient health systems strive to attain [135].

The World Health Organization (WHO) associates the resilience of health systems with health security and universal health coverage; therefore, initiatives to strengthen resilient health systems contribute to a more secure global health environment, ensuring that healthcare is accessible to all at all times [5]. The WHO’s call for nations to develop resilient health systems must be addressed with urgency [136]. Heeding the call to build resilient health systems necessitates a refined understanding of the concept. Resilience is inherently a dynamic notion that must consider the experiences of individual health systems; therefore, its implementation should not be prescriptive but shaped by the specific context [137].

Our review had some limitations. First, we considered only articles published after 1980, an arbitrary cutoff, acknowledging that valuable studies on health system resilience may have been conducted before this date. Second, we recognize that during title and abstract screening, some articles might have been unintentionally excluded.

Our synthesis highlights that resilience in African health systems is best understood through four capacities, preparedness, absorptive capacity, adaptive capacity, and recovery, each supported by specific WHO health system building blocks [138]. Service delivery, human resources, and leadership and governance emerged as the most critical. Practical examples from Namibia, Nigeria, Rwanda, and Uganda illustrate how these capacities are operationalized [112,139–141].

### Contribution to policy

In Uganda, resilience capacities are reinforced by national policies such as the National Action Plan for Health Security, the Integrated Disease Surveillance and Response strategy, and decentralization reforms, which collectively strengthen preparedness, absorptive capacity, adaptive capacity, and recovery. Community Health Strategies further institutionalize Village Health Teams as frontline actors, embedding resilience within local governance structures.

## Conclusion

The description of health system resilience to infectious diseases emphasizes preparedness, absorptive and adaptive capacities, as well as recovery. Our study underscores seven attributes, five of which are thoroughly represented by the dimensions of a resilient health system: awareness, integration, diversity, adaptability, and self-regulation. Our review presents community engagement and health education and promotion as essential attributes of a resilient health system. The implications of these findings are that preparedness needs to be integrated in the definition of health system resilience, highlighting the role of prevention in the concept of resilience. Considering the operational definition of recovery that encompasses bouncing back to pre-shock levels, policymakers should explore the incorporation of lessons learned to strengthen health systems beyond their pre-outbreak state. We recommend these elements of resilience from these findings be included in the frameworks of health system resilience within the continent. Our findings provide the foundation for the development of tools for the measurement and monitoring of health system resilience, on which future research can build.

## Supporting information

S1 Checklist PRISMA Reporting Guidelines, adapted from: Page MJ, McKenzie JE, Bossuyt PM, Boutron I, Hoffmann TC, Mulrow CD, et al. The PRISMA 2020 statement: an updated guideline for reporting systematic reviews. BMJ. 2021;372:n71. https://doi.org/10.1136/bmj.n71. Licensed under Creative Commons Attribution (CC BY 4.0).
(DOCX)

S1 Appendix Search strategy and search log. (DOCX)

S1 Table Excluded articles. (DOCX)
