# Supplementary material for: Resilience of health systems in Africa to infectious disease shocks: A qualitative evidence synthesis
Source: PLOS Glob Public Health. 2026 Aug 7;6(8):e0006938. doi: 10.1371/journal.pgph.0006938 (PMC13450801; doi:10.1371/journal.pgph.0006938)
Supplement: Supplementary file 1 [file pgph.0006938.s001.docx]

Manuscript Number: PGPH-D-26-00417- PLOS Global Public Health

Dear Editor and Authors,

Thank you for the opportunity to review the manuscript titled *“Resilience of health systems in Africa to infectious disease shocks: A qualitative evidence synthesis.”* I appreciate the relevance of this work and have provided concise comments to support strengthening the clarity and rigor of the manuscript. Please find my feedback below.

Kind regards,
**Akalewold Gebremeskel**

**General Comment:**
Thank you for the opportunity to review this manuscript. I read it with great interest, as the topic aligns closely with my own work. I appreciate the importance and relevance of the study; however, I have concerns regarding the methodological clarity and rigor. Strengthening the methodological justification and ensuring a more convincing presentation of the analytic process would enhance the overall credibility of the paper. The analysis could be strengthened by aligning it more clearly with the WHO Health System Framework as a lens for examining system attributes. *Financial capacity* is appropriately considered.

**See my Specific Feedback below:**

**L76:**
Please use commonly accepted and current terminology. For example, instead of “in developing countries,” consider using terms such as *“low‑ and middle‑income countries (LMICs)”* or *“low‑resource settings.”* These terms are widely used by the UN, World Bank, WHO, and contemporary scholarly literature.

**L78–79:**
Please provide concrete examples to support the statement that “multiple outbreaks are frequently observed.” For instance:
*“Since 2020, the African region has faced more than XX major outbreaks annually, and Uganda alone experienced over 800 between 2000 and 2022.”* (13,14)

**Introduction:**
The introduction should more clearly highlight the *gap*, specifically the “lack of understanding,” and explain how this lack of understanding has contributed to the current health system challenges in Africa.

**Method Section**

**SPIDER Framework**

Could you please clarify how the SPIDER framework was applied in developing your search strategy? It would be helpful to outline:

- How the research question was identified.
- How keywords were generated for each SPIDER component (Sample, Phenomenon of Interest, Design, Evaluation, Research Type).
- How these terms were translated into database‑ready search strings.
- How SPIDER blocks were constructed and combined into the final search strategy.
- How the strategy was adapted for each database (e.g., use of MeSH terms in PubMed).

**Search Management Workflow**

The workflow for managing search results is not fully clear. Please clarify:

- Why Zotero was used for duplicate removal,
- Why Rayyan was used for title/abstract screening,
- Why Covidence was selected for full‑text screening.

It would be helpful to explain why a multi‑platform approach was preferred instead of using a single integrated system (e.g., Rayyan alone or Covidence alone). Please elaborate on how these tools complemented one another.

**L142–144:**

Please clarify whether you conducted any manual or supplementary searches when full texts were not accessible through the databases.
For example:

- Searches on Google Scholar, publisher websites, ResearchGate, or institutional repositories
- Contacting authors for missing full texts.

**Line 194: Data Management and Analysis**

The analytic procedures require further clarification to assess rigor and transparency. Please specify the exact steps followed in conducting the inductive thematic analysis, including:

- How initial codes were generated (e.g., line‑by‑line, open coding).
- How codes were grouped into categories and what rules or criteria guided categorization.
- How the WHO framework informed your analytic process (e.g., during coding, theme development, or interpretation).
- Whether Framework Analysis was considered, given the use of an existing framework, and why inductive thematic analysis was chosen instead.
- How NVivo supported your analysis (e.g., setting up nodes, coding procedures, queries, matrix coding, memoing) and the sequence of steps followed.

**Results Section**

**L206:**
Please state the exact names or number of databases used instead of saying “various electronic databases.”

**Line 223:**
The subtitle *“Categories generated from themes”* could be improved, as themes are typically the result of the categorization process. A more accurate phrasing could clarify the flow from *codes → categories → themes*.

You could also highlight the attributes of resilience and explain why some WHO health system pillars were not represented—for example, because the included studies did not cover those areas.

**Discussion Section**

- The different aspects of resilience need clearer and more specific descriptions.
- Align the discussion more explicitly with the WHO Health System Framework.
- Providing practical examples or case studies illustrating *preparedness, absorptive capacity, adaptive capacity,* and *recovery* would strengthen the definition of a resilient health system.
- It would also be helpful to highlight which WHO health system building blocks are most critical for strengthening resilience in African health systems.
- Add specific policy aspects
