# Supplementary material for: Resilience of health systems in Africa to infectious disease shocks: A qualitative evidence synthesis
Source: PLOS Glob Public Health. 2026 Aug 7;6(8):e0006938. doi: 10.1371/journal.pgph.0006938 (PMC13450801; doi:10.1371/journal.pgph.0006938)
Supplement: Supplementary file 2 [file pgph.0006938.s002.docx]

Denis Okethwangu
Makerere University School of Public Health
Mulago Hospital Complex

Kampala

[dokethwangu@musph.ac.ug](mailto:dokethwangu@musph.ac.ug)

January 31, 2026

The Editor
PLOS Global Public Health,

Subject: Response to concerns raised regarding manuscript PGPH-D-26-00267

Thank you for your response to our manuscript submission and for the opportunity to revise our paper on **‘Resilience of health systems in Africa to infectious disease shocks: A qualitative evidence synthesis’**. The Editor raised concerns about the exclusion of non-English articles. As such, we edited our exclusion list, removing non-English articles, and translated the non-English articles, which had previously been excluded. This is indicated in the manuscript on Lines 156 to 160 under the Methods section.

We hope the recent version of the manuscript is more acceptable to PLOS Global Public Health, and we remain available for any clarification.

Sincerely,

Denis Okethwangu
