# Supplementary material for: Resilience of health systems in Africa to infectious disease shocks: A qualitative evidence synthesis
Source: PLOS Glob Public Health. 2026 Aug 7;6(8):e0006938. doi: 10.1371/journal.pgph.0006938 (PMC13450801; doi:10.1371/journal.pgph.0006938)
Supplement: Supplementary file 3 [file pgph.0006938.s003.docx]

**Revised Review Feedback**

Thank you for addressing the comments I previously provided. I can see that there has been improvement in the manuscript.

However, I am still not fully convinced regarding my previous comment on **#1 (Search Management Workflow)**.

The workflow for managing search results is not fully clear. Please clarify: • Why Zotero was used for duplicate removal, • Why Rayyan was used for title/abstract screening, • Why Covidence was selected for full text screening. It would be helpful to explain why a multi platform approach was preferred instead of using a single integrated system (e.g., Rayyan alone or Covidence alone). Please elaborate on how these tools complemented one another.

If Rayyan was used for title/abstract screening and then articles were transferred to Covidence for full-text screening, this transition should be explicitly described in the methods section for **transparency and clarity**.

Additionally, since both Rayyan and Covidence have duplicate detection capabilities, it is unclear why Zotero was required for this step. Please justify its inclusion in the workflow. If retained, Zotero should be clearly described as a **reference management tool**, with its specific role clearly articulated.

**#2 Reference Duplications**

Please double-check the references for duplication. For example:

- References **17 and 46** appear to be duplicates,
- References **87 and 93** also appear to be duplicates.

**#3 Overall Clarity and Coherence**

The manuscript would benefit from a thorough review to improve clarity, coherence, and logical flow. Strengthening the organization and transitions between sections will help ensure that the audience can clearly follow the argument and methodology. Additionally, attention should be given to maintaining academic rigor and consistency in writing.
