# Supplementary material for: Resilience of health systems in Africa to infectious disease shocks: A qualitative evidence synthesis
Source: PLOS Glob Public Health. 2026 Aug 7;6(8):e0006938. doi: 10.1371/journal.pgph.0006938 (PMC13450801; doi:10.1371/journal.pgph.0006938)
Supplement: Supplementary file 4 [file pgph.0006938.s004.docx]

Denis Okethwangu
Makerere University School of Public Health
Mulago Hospital Complex

Kampala

[dokethwangu@musph.ac.ug](mailto:dokethwangu@musph.ac.ug)

April 18, 2026

The Editor
PLOS Global Public Health,

Subject: Response to reviewer comments on manuscript PGPH-D-26-00417

Thank you for your response to our manuscript submission and for the opportunity to improve our paper on **‘Resilience of health systems in Africa to infectious disease shocks: A qualitative evidence synthesis’**.

Here below are my point-by-point responses to each of the reviewer’s comments (italicized, in bold red font). I appreciate the comments and do hope that they have been satisfactorily addressed

We hope the recent version of the manuscript is more acceptable to PLOS Global Public Health, and we remain available for any clarification.

Sincerely,

Denis Okethwangu

**General Comment:**
Thank you for the opportunity to review this manuscript. I read it with great interest, as the topic aligns closely with my own work. I appreciate the importance and relevance of the study; however, I have concerns regarding the methodological clarity and rigor. Strengthening the methodological justification and ensuring a more convincing presentation of the analytic process would enhance the overall credibility of the paper. The analysis could be strengthened by aligning it more clearly with the WHO Health System Framework as a lens for examining system attributes. *Financial capacity* is appropriately considered.

**See my Specific Feedback below:**

**L76:**
Please use commonly accepted and current terminology. For example, instead of “in developing countries,” consider using terms such as *“low‑ and middle‑income countries (LMICs)”* or *“low‑resource settings.”* These terms are widely used by the UN, World Bank, WHO, and contemporary scholarly literature.

***We appreciate this comment, and we have edited this in Line 76.***

**L78–79:**
Please provide concrete examples to support the statement that “multiple outbreaks are frequently observed.” For instance:
*“Since 2020, the African region has faced more than XX major outbreaks annually, and Uganda alone experienced over 800 between 2000 and 2022.”* (13,14)

***The original statement had the specific number of outbreaks, as follows: “Since 2020, the African region has faced more than 100 major outbreaks annually; Uganda alone experienced over 800 between 2000 and 2022”. However, we have also added a more specific citation relevant to only Uganda. This is on Lines 80-82.***

**Introduction:**
The introduction should more clearly highlight the *gap*, specifically the “lack of understanding,” and explain how this lack of understanding has contributed to the current health system challenges in Africa.

***We have made improvements to the gap, and now it reads: There is currently a limited shared understanding of resilience, causing delays in operationalization of the concept to strengthen systems. Resilience has often been narrowed to emergency response rather than to the broader aspects that sustain essential services, including financing, human resources, and community engagement (line 93).***

**Method Section**

**SPIDER Framework**

Could you please clarify how the SPIDER framework was applied in developing your search strategy? It would be helpful to outline:

- How the research question was identified.

***We have indicated in Lines 131-134 how we used SPIDER to formulate our research question. We have also included a table in the attached Supporting Information, S1 Text.***

- How keywords were generated for each SPIDER component (Sample, Phenomenon of Interest, Design, Evaluation, Research Type).

***S1 Text, which is supplementary information attached to the document indicates how keywords were generated for each SPIDER component.***

- How these terms were translated into database‑ready search strings.

***In our manuscript, lines 121 to 123, we indicated how these terms were translated into database-ready search strings. It reads: “Using Boolean operators and truncations, we adopted the search terms from PubMed for the other databases depending on the limits to number of search terms and unique search features”.***

- How SPIDER blocks were constructed and combined into the final search strategy.

***In our manuscript, on Line 119, we indicate that we combined the different keywords to formulate a strategy, initially for PubMed but later adapted for the other databases too. We have included a sentence in parentheses on Lines 119 to 120 directing the reader to the supporting material.***

- How the strategy was adapted for each database (e.g., use of MeSH terms in PubMed).

***This is indicated in great detail in S1 Text, the supporting information, which we direct our readers to on Lines 143 to 144. I hope you find this agreeable.***

**Search Management Workflow**

The workflow for managing search results is not fully clear. Please clarify:

- Why Zotero was used for duplicate removal,

***On Lines 146 and 147, we indicate the reason for our choice of Zotero for duplicate removal. The Corresponding Author (also first author) DO is a long-time user of Zotero and finds it very customizable for such functions.***

- Why Rayyan was used for title/abstract screening,

***Rayyan was our application of choice for the review. I was initially impressed by its basic functionalities and that it was free. We used it for title/abstract screening, and hoped to use it for all the subsequent steps too.***

- Why Covidence was selected for full‑text screening

***However, we later found out that, for our work, Covidence was a better option. We then paid subscription for Covidence and made the switch from Rayyan.***

It would be helpful to explain why a multi‑platform approach was preferred instead of using a single integrated system (e.g., Rayyan alone or Covidence alone). Please elaborate on how these tools complemented one another.

***Our intention was never to use the two platforms. The decision, as described above, was made during the process of conducting the review.***

**L142–144:**

Please clarify whether you conducted any manual or supplementary searches when full texts were not accessible through the databases.
For example:

- Searches on Google Scholar, publisher websites, ResearchGate, or institutional repositories

***We relied on institutional subscriptions from Makerere University and the Norwegian Institute of Public Health to access articles, which were accessible behind paywalls***

- Contacting authors for missing full texts

***Yes, we did write emails to authors of papers we were not able to access (where their addresses were available), but none of the emails were responded to.***

**Line 194: Data Management and Analysis**

The analytic procedures require further clarification to assess rigor and transparency. Please specify the exact steps followed in conducting the inductive thematic analysis, including:

- How initial codes were generated (e.g., line‑by‑line, open coding).

***We have now included line-for-line coding on Line 208.***

- How codes were grouped into categories and what rules or criteria guided categorization.

***On Lines 208 to 211, we indicate the criteria that guided categorization of generated codes.***

- How the WHO framework informed your analytic process (e.g., during coding, theme development, or interpretation)

***Our analysis did not build on any theories or frameworks; we allowed themes to emerge from the generated codes.***

- Whether Framework Analysis was considered, given the use of an existing framework, and why inductive thematic analysis was chosen instead

***While we collected data on the WHO building blocks of the health system, our analysis of the codes did not take them into account. The analysis of the building blocks stopped at descriptive analysis as indicated on Lines 222 to 223.***

- How NVivo supported your analysis (e.g., setting up nodes, coding procedures, queries, matrix coding, memoing) and the sequence of steps followed.

***We have included in the manuscript how NVivo supported our qualitative analysis. This may be found in Lines 212 to 219. I hope you find it satisfactory.***

**Results Section**

**L206:**
Please state the exact names or number of databases used instead of saying “various electronic databases.”

***Thank you for this comment. This has now been improved in the text, on Line 230.***

**Line 223:**
The subtitle *“Categories generated from themes”* could be improved, as themes are typically the result of the categorization process. A more accurate phrasing could clarify the flow from *codes → categories → themes*.

***The subtitle has been changed to “Capacities generated from themes” from “Categories generated from themes”, which we believe was confusing considering the flow, which you clearly highlighted above.***

You could also highlight the attributes of resilience and explain why some WHO health system pillars were not represented—for example, because the included studies did not cover those areas.

***As mentioned earlier, during coding and analysis of data, we did not set out to build onto any theories or frameworks. The attributes of resilient health systems that resulted were those that emerged from the data that we extracted. We described each of them in the Results section, and in the Discussion section (from Line 411) speak about them in relation to other published work, including the WHO frameworks.***

**Discussion Section**

- The different aspects of resilience need clearer and more specific descriptions.

***We have included more refined definitions of the emergent aspects of resilience to infectious disease shocks from Lines 386 to 396. We hoped that this would provide more clarity.***

- Align the discussion more explicitly with the WHO Health System Framework.

***Our discussion of the definition of health system resilience is made relative to relevant literature, including those from WHO. However, we felt that leaning towards any one particular framework or theory would cage our work and give it a narrow perspective. While we drew from the WHO Health System Framework, we felt that our discussion should take a broader lens.***

- Providing practical examples or case studies illustrating *preparedness, absorptive capacity, adaptive capacity,* and *recovery* would strengthen the definition of a resilient health system.

***In wrapping up the Discussion section, we pick out countries with practical examples that illustrate the different aspects of resilience. I hope this is what you meant and that you will find our method agreeable.***

- It would also be helpful to highlight which WHO health system building blocks are most critical for strengthening resilience in African health systems.

***As demonstrated in our results, we highlight in Lines 271 and 274 the building blocks that were most represented in the records reviewed.***

- Add specific policy aspects

***From Lines 491 to 495, we attempt to include specific policy aspects that are relevant to our work.***
