# Supplementary material for: Resilience of health systems in Africa to infectious disease shocks: A qualitative evidence synthesis
Source: PLOS Glob Public Health. 2026 Aug 7;6(8):e0006938. doi: 10.1371/journal.pgph.0006938 (PMC13450801; doi:10.1371/journal.pgph.0006938)
Supplement: Supplementary file 5 [file pgph.0006938.s005.docx]

**General Comment:**

Thank you for addressing the comments I previously provided. I can see that there has been an improvement in the manuscript. However, I am still not fully convinced regarding my previous comment on **#1 (Search Management Workflow).**

The workflow for managing search results is not fully clear. Please clarify: • Why Zotero was used for duplicate removal, • Why Rayyan was used for title/abstract screening, • Why Covidence was selected for full text screening. It would be helpful to explain why a multi-platform approach was preferred instead of using a single integrated system (e.g., Rayyan alone or Covidence alone). Please elaborate on how these tools complemented one another.

If Rayyan was used for title/abstract screening and then articles were transferred to Covidence for full-text screening, this transition should be explicitly described in the methods section for **transparency and clarity**.

Additionally, since both Rayyan and Covidence have duplicate detection capabilities, it is unclear why Zotero was required for this step. Please justify its inclusion in the workflow. If retained, Zotero should be clearly described as a **reference management tool**, with its specific role clearly articulated.

***We have clarified the workflow and rationale for tool selection in the revised manuscript. The transition between platforms has now been explicitly described in the Methods section for transparency. These are included in lines 147 to 156 of the clean version of the revised manuscript.***

***Zotero was used as a reference management tool to perform duplicate removal prior to screening. While both Rayyan and Covidence include deduplication functions, Zotero provides a more structured system for identifying and merging duplicates, particularly when handling larger datasets. For this reason, all records were first exported to Zotero for deduplication.***

***Rayyan was used for title and abstract screening due to its suitability for rapid screening and its user-friendly interface. Following completion of this phase, all studies meeting inclusion criteria were exported from Rayyan and imported into Covidence.***

***Covidence was selected for full-text screening because of its more advanced functionality for managing later stages of the review, including structured workflows and study selection processes.***

***The use of multiple platforms was primarily driven by practical considerations and the complementary strengths of each tool: Zotero for reliable duplicate removal, Rayyan for efficient initial screening, and Covidence for comprehensive full-text review. The transition between tools was straightforward and did not affect the integrity of the review process.***

#2 Reference Duplications

Please double-check the references for duplication. For example:

•	References 17 and 46 appear to be duplicates,

•	References 87 and 93 also appear to be duplicates.

***Thank you very much for pointing this out. We have confirmed that both cases were duplicates and have rectified the anomaly.***

#3 Overall Clarity and Coherence

The manuscript would benefit from a thorough review to improve clarity, coherence, and logical flow. Strengthening the organization and transitions between sections will help ensure that the audience can clearly follow the argument and methodology. Additionally, attention should be given to maintaining academic rigor and consistency in writing.

***We have conducted a thorough review of the manuscript and made some improvements to enhance its flow. We sincerely hope that the manuscript has improved in clarity, coherence, and flow.***
